# Benefits of Ion Mobility Separation in GC-APCI-HRMS Screening: From the Construction of a CCS Library to the Application to Real-World Samples

**DOI:** 10.1021/acs.analchem.2c01118

**Published:** 2022-06-13

**Authors:** David Izquierdo-Sandoval, David Fabregat-Safont, Leticia Lacalle-Bergeron, Juan V. Sancho, Félix Hernández, Tania Portoles

**Affiliations:** Environmental and Public Health Analytical Chemistry, Research Institute for Pesticides and Water (IUPA), University Jaume I, Av. Sos Baynat S/N, 12071 Castellón de la Plana, Spain

## Abstract

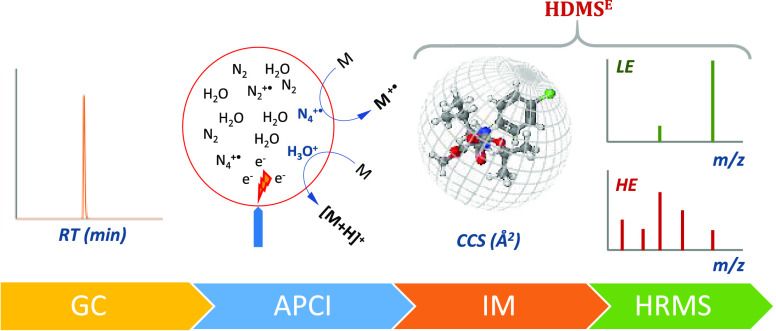

The performance of gas chromatography (GC) combined with the improved identification properties of ion mobility separation coupled to high-resolution mass spectrometry (IMS-HRMS) is presented as a promising approach for the monitoring of (semi)volatile compounds in complex matrices. The soft ionization promoted by an atmospheric pressure chemical ionization (APCI) source designed for GC preserves the molecular and/or quasi-molecular ion information enabling a rapid, sensitive, and efficient wide-scope screening. Additionally, ion mobility separation (IMS) separates species of interest from coeluting matrix interferences and/or resolves isomers based on their charge, shape, and size, making IMS-derived collision cross section (CCS) a robust and matrix-independent parameter comparable between instruments. In this way, GC-APCI-IMS-HRMS becomes a powerful approach for both target and suspect screening due to the improvements in (tentative) identifications. In this work, mobility data for 264 relevant multiclass organic pollutants in environmental and food-safety fields were collected by coupling GC-APCI with IMS-HRMS, generating CCS information for molecular ion and/or protonated molecules and some in-source fragments. The identification power of GC-APCI-IMS-HRMS for the studied compounds was assessed in complex-matrix samples, including fish feed extracts, surface waters, and different fruit and vegetable samples.

## Introduction

Gas chromatography (GC) coupled to high-resolution mass spectrometry (HRMS) is a powerful analytical technique for monitoring nonpolar, volatile, and thermally stable organic pollutants in a wide variety of samples.^1^ HRMS analyzers, such as time of flight (TOF) or Orbitrap, are able to provide accurate-mass full-spectrum acquisition data with reasonable sensitivity, making possible the screening for a virtually unlimited number of substances in a single analysis.^2^ TOF-MS with an electron ionization (EI) source is widely implemented for the screening of broad lists of GC-amenable compounds.^3,4^ The applicability of orbitrap, also with an EI source, is gaining popularity due to its higher resolving power [around 120,000 full width at half-maximum (FWHM)] and its consistency in terms of mass accuracy (≤1 mDa).^5,6^ For GC, EI is currently the most implemented ionization technique due to its robustness, reproducibility, and the amount of mass spectral data available in commercial libraries.^7^ However, the EI extensive in-source fragmentation and the highly probable absence of the molecular ion is a drawback for suspect screening or “nontarget” purposes as it is difficult to predict which will be the most abundant fragment ions of the compounds.^1^

In contrast to EI, the soft ionization promoted by atmospheric pressure chemical ionization (APCI) preserves the molecular and/or quasi-molecular ion information, enhancing the sensitivity and selectivity of the MS, thus making data processing for wide-scope screening easier and more efficient.^8,9^ The identification potential is considerably increased by the use of hybrid HRMS mass analyzers, such as quadrupole-time of flight (QTOF), allowing for the acquisition of information about ionized molecules and fragment ions in a single injection.^10,11^ Despite the benefits of APCI in GC–HRMS-based screening, there are some drawbacks that limit its applicability on a large scale. APCI is quite condition-dependent (flow conditions, source geometry, cone voltages, humidity, temperature, etc.), which may have a negative impact on the reproducibility;^12^ moreover, the shortage of GC-APCI-HRMS-based spectra in available databases is also a limiting factor for a larger implementation of this technique.^7,13^ A possible way to improve the comparability of spectra, as well as the identification power of the screening and the handling of complex mixtures is the coupling of GC-APCI with ion mobility and HRMS.^12,14^

The development of new HRMS instruments equipped with ion mobility separation (IMS-HRMS) has emerged as a powerful tool for target, suspect, and nontarget screening in the last years.^15−17^ These instruments measure drift time (DT) in the IMS cell and provide collisional-cross section (CCS) values that can be used as an additional identification parameter, as well as to obtain cleaner spectra based on DT alignment.^18−20^ The use of drift-aligned fragment spectra for a specific precursor allows the separation/removal of fragment ions coming from matrix interferents, making the mass spectrum interpretation easier.^21^ The CCS parameter, expressed in Å^2^, is not affected by the complexity of the matrix and is partially orthogonal to other molecular indicators such as chromatographic retention time (RT), *m/z* ratio, isotopic pattern and fragment ions, becoming an interesting item to be included in mass spectra databases.^18,22,23^ CCS values have been empirically reported to be comparable between different instruments and experimental conditions, with deviations within ±2% when using instruments with the same IMS technology.^24−26^ Moreover, the CCS prediction based on machine learning tools for small molecules, such as pharmaceuticals, pesticides, and drugs of abuse, has demonstrated its usefulness for suspect screening purposes.^27,28^

Despite the large number of applications historically reported upon the use of IMS as detector for GC,^29^ the inclusion of IMS as an additional ion separator within GC–HRMS systems is still in early stages.^30^ To the best of our knowledge, only a few applications of GC-APCI coupled with IMS-HRMS have been reported for real-world cases.^31−34^ In one such application, a comprehensive two-dimensional GC system (GCxGC) coupled with drift-tube IMS (DTIMS)-QTOF was developed for the screening of organic pollutants, enhancing the identification power by using a home-made CCS database of drug-like compounds and pesticides previously built for LC-electrospray-MS applications.^31^ The authors stated the lack of CCS data for GC-amenable compounds, which was a limitation on the number of possible hits during the identification step, and claimed the need for expanding the CCS database for a more powerful screening. Considering the scarcity of mobility data available, any contribution exploring the ion mobility behavior of volatile and semivolatile compounds is highly relevant. In this line, Zheng et al. collected CCS for 120 standards, including polycyclic aromatic hydrocarbons (PAHs), polychlorinated biphenyls (PCBs), polybrominated diphenyl ethers (PDBEs), and their hydroxylated metabolites, by employing different ionization sources (APCI included), in a DTIMS-QTOF MS. In that work, the potential of this technique to separate isomers in each xenobiotic class was emphasized.^32^ Recently, the use of a GC-APCI-IMS-QTOF-MS instrument equipped with traveling wave IMS (TWIMS) has been reported to enhance the identification power for the characterization of fatty acid methyl esters in edible oils^33^ and long-chain polyunsaturated fatty acids in fish.^34^

In the present work, we investigate the potential of GC-APCI-IMS-QTOF MS for the screening of organic contaminants in complex-matrix samples and illustrate such potential with key examples of real-world applications. To this aim, mobility data for 264 GC-amenable compounds have been collected by using standards to get accurate CCS values to build a home-made CCS database. The compounds selected included pesticides, PAHs, polychlorinated biphenyls (PCBs), flame retardants (brominated and phosphonated), and different emerging contaminants, such as insect repellents, musks, and UV-filters among others. The data collected comprised CCS values for molecular ions and/or protonated molecules and in-source fragments, favoring the formation of one ion or another in the source. Information provided in this work will be of help for future target and suspect GC-IMS-HRMS screening applications in applied fields, such as environmental pollution, food safety, or toxicology, among others.

## Experimental Section

### Chemical and Materials

A total of 264 reference standards, purchased from different vendors, including 18 PCBs, 14 brominated flame retardants (BFRs), 16 organophosphate flame retardants (OPFRs), 23 PAHs, 182 multiclass pesticides, and 11 emerging pollutants including insect repellents, musks, and UV-filters among others, were injected for the development of an in-house library for screening purposes in GC-APCI-IMS-QTOF (more details in the Supporting Information).

### Samples Selected as Case Study

Different types of samples were selected to illustrate the benefits of GC-APCI-IMS-HRMS in the wide-scope screening of organic micropollutants: river water (RW), fish feed, and fruit and vegetable commodities. Four surface water samples were collected in the lower section and the estuary of the Mijares river in Eastern Spain. The sampling points correspond to the sites 16, 17, 18, and 19 selected by Bijlsma et al.^35^ Samples were collected in polyethylene bottles, transported in refrigerated isothermal containers and stored in the dark at −20 °C until their analysis. Plant-based fish feed (wheat gluten) was provided by Biomar (Gragemouth, UK), while fruit and vegetable commodities were purchased from a local food store in Castelló (Spain).

RW extraction and preconcentration was made by solid-phase extraction (SPE) based on the method developed by Bijlsma et al.,^35^ using a mixed-mode stationary phase (Oasis HLB, Waters) with a preconcentration factor ×1000. Sample preparation methods for fish feed, fruits, and vegetables samples were based on different versions of QuEChERS approaches previously applied in our laboratory.^36,37^ Quality controls (QCs) at several concentration levels were prepared for representative matrices. (Further information about sample preparation can be found in the Supporting Information).

### Instrumentation

Analyses were performed with an Agilent 7890N gas chromatograph (Palo Alto, CA, USA) equipped with an Agilent 7693 autosampler. The gas chromatograph was interfaced to a VION IMS-QTOF mass spectrometer (Waters Corporation, Manchester, UK) employing APGC v2.0 as the ionization source, working in positive APCI mode.

GC separation was achieved by the use of a fused silica DB-5MS capillary column with a length of 30 m × 0.25 mm i.d. and a film thickness of 0.25 μm (J&W Scientific, Folson, CA, USA). A total runtime of 50 min was set up following the temperature program: 90 °C (1 min); 5 °C/min to 315 °C; 4 min hold. Pulsed splitless (30 psi) injections of 1 μL were carried out at 280 °C with a splitless time of 1 min. Helium 99.999% (Praxair, Spain) was used as carrier gas at a flow of 4 mL/min.

The APCI corona discharge pin was operated at 2.0 μA and the cone voltage was set to 20 V. The interface and ionization source temperatures were set to 325 and 150 °C, respectively. N_2_ was used as auxiliary gas at 300 L/h, as cone gas at 160 L/h and as make-up gas at 275 mL/min.

MS data were acquired by a TWIMS-QTOF MS in high-definition (HD) MS^E^ mode, in the range 50–1000 *m/z*, with N_2_ as the drift gas, an IMS wave velocity of 250 m/s, and wave height ramp of 20–50 V. Two independent acquisition functions with different collision energies were acquired during the run: a collision energy of 6 eV for the low energy function (LE), and a ramp of 21–56 eV for the high energy function (HE). HDMS^E^ implies DT alignment between LE and high energy (HE) spectra keeping only fragment ions related to parent ions. The scan times for both LE and HE functions were 0.25 s. Nitrogen (≥99.999%) was used as collision-induced dissociation (CID) gas.

Internal mass calibration was performed using two GC-column bleeding ions as lock mass (monitoring the molecular ions, *m/z* 355.06693 and 223.06365 corresponding to decamethylcyclopentasiloxane and hexamethylcyclotrisiloxane, respectively) and octafluoronaphthalene (m/z 271.98668) for analysis of food matrices. The instrument was calibrated for both *m/z* measurements and CCS calculation following the manufacturer’s instructions using a Z-Spray electrospray ionization source (Waters Corp.).

In order to enhance proton transfer ionization, a vial filled with water and closed with aluminum foil in which small perforations were made, was placed in a designed holder into the APCI source door to enhance protonation (wet conditions). For dry conditions, the APCI source was maintained at 150 °C overnight prior to analysis, in order to remove water traces. MS data were acquired and processed using UNIFI informatics platform (v 1.9) from Waters.

### Screening of GC-Amenable Compounds in Real-World Samples

Target screening was performed using the in-house database developed in this work. The database included information about RT, mass spectrometric data and CCS values for molecular ions and protonated molecules of 264 GC-amenable organic pollutants. The automated workflow and identification criteria proposed by Celma et al.^38^ for the LC-IMS-HRMS screening of organic pollutants in environmental samples was followed in the current work. Briefly, GC/LC-IMS-HRMS systems generate a 4-dimensional data set: (1) RT, (2) drift time (DT), (3) accurate mass, and (4) intensity. These parameters enable the alignment of the molecular or quasi-molecular ion, commonly observed in LE spectra, with its fragments from the HE spectra in terms of RT and DT. To reach the highest level of identification reliability (i.e., confirmation) (level 1) the following requirements must be accomplished: mass accuracy of both precursor and fragment ions *<*5 ppm, RT deviation *<*0.1 min, and CCS deviation *<*2% from the reference standard value.

## Results and Discussion

### GC-APCI-TWIMS-HRMS Library

APCI source coupled to GC is known to generate two main ionization mechanisms: charge transfer and proton transfer.^8^ For those analytes whose ionization potential is lower than the ionization potential of the reagent gas, normally nitrogen, change-transfer is usually the main mechanism, producing the molecular ion M^+•^. Those compounds with relatively high proton affinity are prone to generate protonated molecules [M + H]^+^ due to the proton transfer reactions with the hydronium produced by the nitrogen plasma ions normally in wet conditions. Which mechanism occurs depends mainly on the chemical structure of the compound and source atmosphere environment.^7^ It has also been reported that some compounds are not capable of producing stable ions in any of the mentioned mechanisms, as they are fragmented directly at the source.^39^

For the development of the compound library and selection of the optimal measurement conditions, a comprehensive study in terms of signal, in-source fragmentation and ionization mechanism was performed for each compound included in the library, in both dry and wet conditions. Depending upon the fragmentation/ionization behavior, compounds were divided into two different lists, one for each ionization mechanism. The aim of this distribution was to facilitate and expedite the rapid screening of the compounds, considering the species that provide the best sensitivities in the appropriate ionization mode. In those cases where charge transfer and protonation occurred simultaneously in dry conditions, such as with PAHs, sensitivity for M^+•^ in dry and [M + H]^+^ in wet conditions were compared. For those compounds in which neither species showed a substantial response, in-source fragmentation was examined.

With the above categories, the library ultimately contained a total of 110 compounds for charge transfer conditions, 91 for which M^+•^ was selected, and 19 where the selection was as the in-source fragment. The list for proton transfer conditions included a total of 154 compounds, 145 compounds selected as [M + H]^+^ and 9 as in-source fragments. The complete lists of compounds in the dry and wet libraries can be found in Tables S1 and S2, respectively. The information provided includes CCS values and fragment ions obtained in the HE spectra (HDMS^E^) of the selected ionized species. The empirical value of CCS was established by averaging the six values obtained after triplicate injection of the standard mixtures at the two different concentration levels. Only the highest level was considered in those cases where no signal was observed at the lowest level.

### CCS Insights for GC-Amenable Compounds in GC-APCI-IMS-HRMS

#### Precision of CCS Measurements

The empirical CCS values included 202 compounds as [M + H]^+^ and 168 as M^+•^. The results obtained for precision, in terms of %RSD, for all ionic species showed an appropriate repeatability among measurements. Precision values were under 0.3% for 93% of [M + H]^+^, 87% of M^+•^ and 96% for in-source fragment ions, and 99% of all ionic species were under 0.5% as seen in Figure S1. No correlation between RSD and standard concentration, or between %RSD and CCS values, were observed. Figure S1A shows the general precision for all ionic species, while Figure S1B shows specific values for 50 randomly selected pesticides. The excellent precision found is in accordance with the results obtained working in electrospray (ESI) for IMS-HRMS^24^ and LC-IMS-HRMS^18,40,41^ suggesting that precision in CCS values is not ionization but mobility-dependent.

#### Orthogonality of CCS against Molecular Mass

One of the strengths of incorporating IMS to MS instruments is the inclusion of an additional parameter complementary to other molecular indicators commonly used for the identification of species, such as *m/z* or chromatographic RT. Apart from obtaining cleaner LE and HE spectra by separating coeluting ionizable compounds, IMS allows the separation of certain isobaric species that present important differences in their CCS values. In this way, CCS becomes an additional parameter available in wide-scope screening analyses for compound identification, reducing the number of potential false positives.

**Figure 1:**
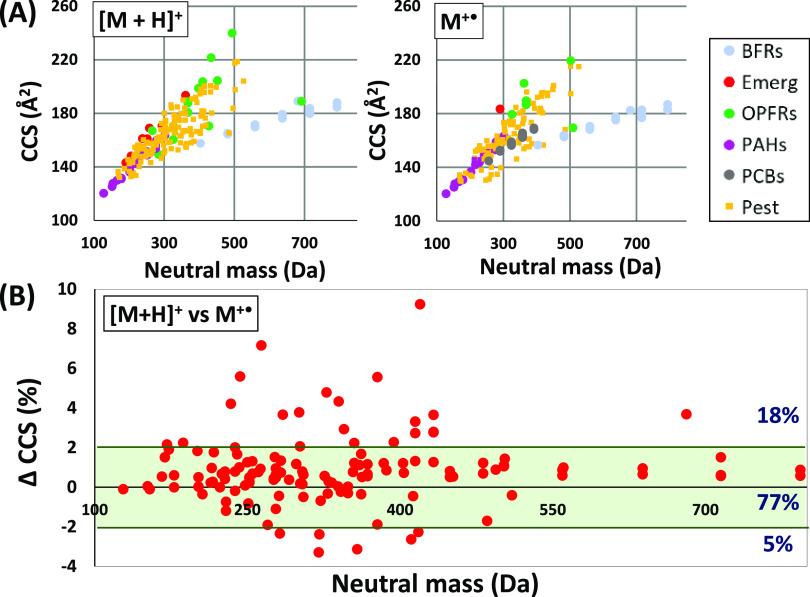
(A) CCS values (Å^2^) versus the neutral mass of the molecule (in Da), for both proton transfer conditions (left) and charge transfer conditions (right). The acronyms refer to: brominated flame retardants (BFRs), emerging pollutants (Emerg), polycyclic aromatic hydrocarbons (PAHs), polychlorinated biphenyl (PCBs), multiclass pesticides (Pest) and organophosphate flame retardants (OPFRs). (B) Deviation in percentage of CCS values observed in compounds showing [M + H]^+^ and M^+•^ species against the neutral mass of the molecule (in Da). Green lines delimit the ±2% tolerance limits. Percentage of compounds within, over, and under these limits are shown in the right.

Figure 1A shows the CCS value of [M + H]^+^ and M^+•^ species against the neutral mass (in Da) of the molecule. Neutral mass is used instead of *m/z* ratio in order to compare CCS values between different ion species of the same molecule. In spite of the strong relationship between CCS and *m/z*, wide CCS ranges were observed at similar neutral masses since different charge distributions could affect the effective area of the ion that collides with drift gas molecules.^42^ For those families that present the same skeletal structure and have small variations in their moieties, such as PAHs, PCBs or BFRs, the CCS values were highly dependent on the neutral mass. In contrast, the group of pesticides, which presents higher chemodiversity, showed important differences in the measured CCS for compounds with similar molecular mass. If the focus is on the general trend followed by the whole set of ions, generally, all the ions adopt a similar distribution across the plot except for BFRs, which possess higher densities due to the bromine atoms. The same situation happened for tris(2,3-dibromopropyl) phosphate (TDBPP), an organophosphate flame retardant with 6 bromines, plotted along with BFRs in protonated transfer conditions.

#### CCS Variation between Ionized Species

It can be noticed (Figure 1A) that the behavior of the different families of GC-amenable compounds in the mobility cell is quite similar regardless of the ionization mechanism that occurred in the APCI source. The importance of the observed trend lies in the possible comparability of CCS values obtained for protonated molecules and for molecular ions, which allows for building common CCS databases regardless the ionization mechanism used in the analysis. To go deeper into this hypothesis, CCS values of the 135 compounds that form the two different species (molecular ion and protonated molecule) were compared and plotted in Figure 1B. This figure shows the CCS deviation between the two ionic species against the neutral mass, with the ±2% region highlighted as the accepted tolerance in CCS deviation for identification purposes.^18^ Based on the results obtained, the proposed hypothesis was remarkably acceptable for 77% of the compounds tested, which showed a CCS difference between protonated molecule and molecular ion lower than 2%.

**Figure 2:**
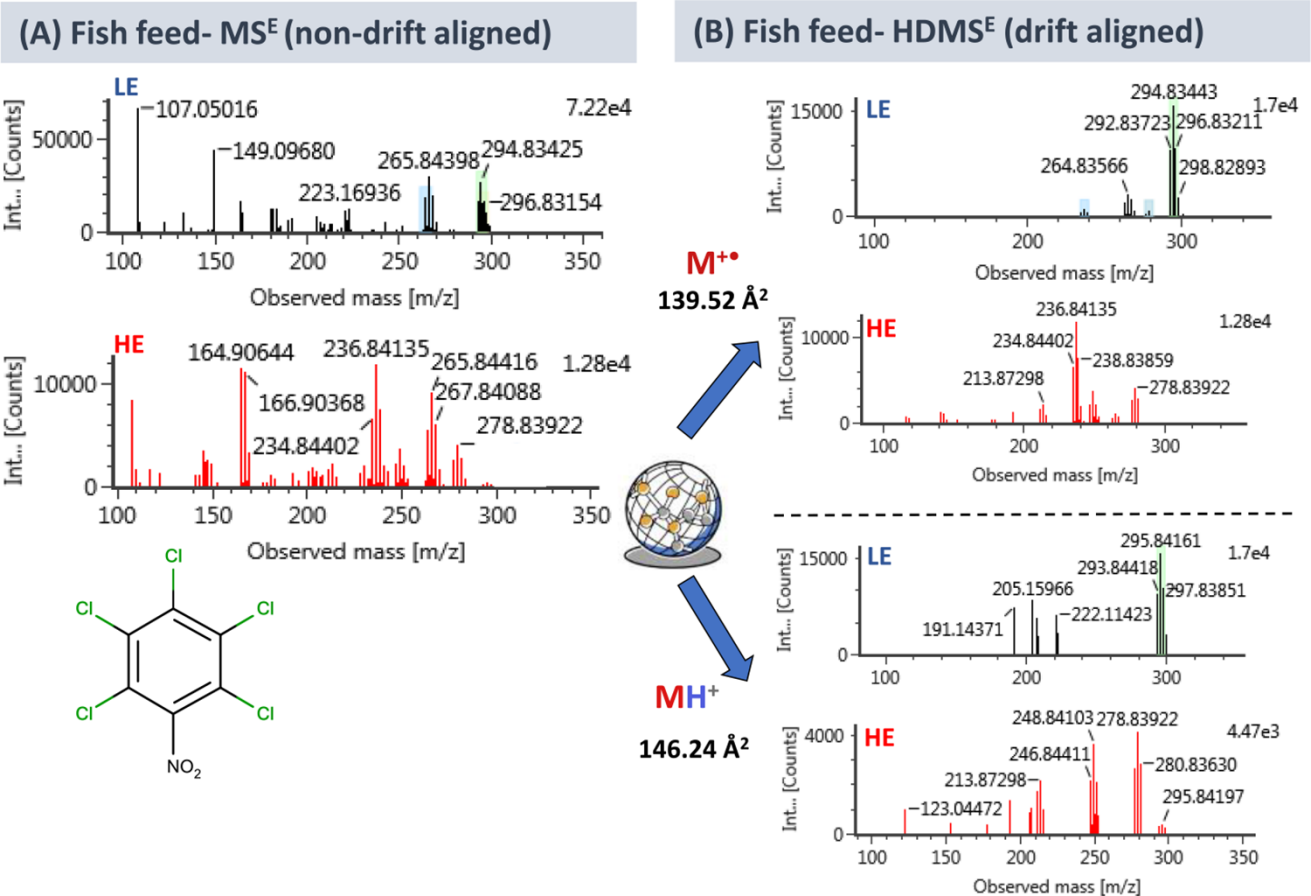
Comparison of HRMS spectra for quintozene in non-DT-aligned data in fish feed sample spiked at 10 μg/L in dry conditions. (A) and in DT-aligned data of the same finding in the same spiked fish feed sample (B). Low energy (LE) and high energy (HE) spectra are shown for both species [M + H]^+^ and M^+•^

However, some compounds showed important CCS deviations between both ionized species. One possible explanation could be the different rearrangements on the structural conformation of the molecule that can be produced depending on the ionization mechanism. Table S3 presents the compounds for which CCS differences between both ionized species was higher than 2%. Interestingly, if the protonated molecule and molecular ion were to be obtained simultaneously, it would be possible to differentiate them as a function of their mobility and feasible to obtain the specific fragmentation spectra of each species. This possibility would be only applicable if both ions present enough separation in the IM cell to allow a successful deconvolution of both mobility peaks. This information can be used to investigate the fragmentation pathways of each ionized species, and is of great interest as an additional identification parameter because two species can be detected for a given compound, providing two CCS values and specific fragment ions for each one. As an example, Figure 2 shows the LE and HE spectra of [M + H]^+^ and M^+•^ for the fungicide pentachloronitrobenzene (quintozene). The empirical CCS values were 146.24 and 139.56 Å^2^ for a [M + H]^+^ and M^+•^, respectively. This difference is significant enough to obtain two separate LE spectra for these species in the IMS-HRMS instrument. As shown in Figure 2B, the fragmentation of each precursor ion was different, as it was observed in the analytical standard solution at the same concentration level (Figure S2), whereas a mixture of both spectra would be obtained without IMS.

The benefits of IMS for mass spectral interpretation is illustrated in Figure 2A,B, which show the spectra for quintozene in a fish feed extract spiked at 10 μg/L with and without DT alignment, respectively. The high complexity of the sample is illustrated by the numerous coeluting compounds that lead to a highly populated spectrum (Figure 2A). Despite such complexity, IMS allowed obtaining LE spectra where [M + H]^+^ and M^+•^ were the base peak, and in which most of the matrix ions were “removed” from spectra (Figure 2B). Additionally, the fragments observed in HE corresponded mostly to the precursor ion species. The presence of matrix ions makes the spectral interpretation difficult, increasing the risk of misidentifications or false positive matchings in fragment databases. IMS adds an extra separation parameter that notably facilitates this process and reduces these errors.

#### Isomers Separation by IMS

The discrimination among isomeric compounds that present differences in their structure, cis/trans configurations and even enantiomers cannot always be achieved by using MS alone.^19^ Some of these isomers can be chromatographically resolved or identified based on the observed fragmentation, but the most challenging ones are those that share similar fragmentation patterns and cannot be separated by GC. As isomeric compounds could present different 3D conformations, and thus different CCS values, IMS provides a new scenario for the identification and separation of these compounds. However, the current resolving power of typical IMS-HRMS instruments still represents a great barrier to achieve this goal, and advanced IMS systems are needed to separate certain types of isomeric compounds.^43^

From the 84 entries with one or more isomers included in this library, only in three cases the obtained ΔCCS was sufficient to discriminate them during screening with the resolution of the IMS instrument used.^44^ However, it is worth pointing out that all of the isomeric compounds included in this study could be chromatographically resolved. Having CCS values as a resource is of particular interest since they have the potential to provide the unambiguous identification of compounds in those cases where chromatographic RT is not available, such as during suspect screening, but where CCS and fragmentation data are available from a compound database.

One of the examples of the pivotal role of IMS is the organophosphate esters trio consisting of the tris (ortho, meta and para)-tolyl phosphate molecules (*m/z* 369.12502, as [M + H]^+^): TOTP (31.05 min | 180.58 Å^2^), TMTP (32.16 min | 187.69 Å^2^) and TPTP (33.48 min | 188.78 Å^2^). Assuming that chromatographic coelution could occur and considering that fragmentation is similar for all of them, TOTP could be discriminated during the screening because the ΔCCS is higher than 2% from the remaining isomers. A similar behavior for these compounds was observed when working with the M^+•^ species. Another interesting example is the pair of regioisomers endrin and dieldrin (*m/z* 378.87791, [M + H]^+^), with CCS in wet conditions of 163.84 and 157.92 Å^2^ (respectively), while in dry conditions are 155.20 and 160.93 Å^2^, respectively. The ΔCCS was slightly higher in proton transfer conditions and both compounds were also more stable in the protonated form; therefore, this configuration could identify these two isomeric organochlorine compounds using CCS.

**Figure 3:**
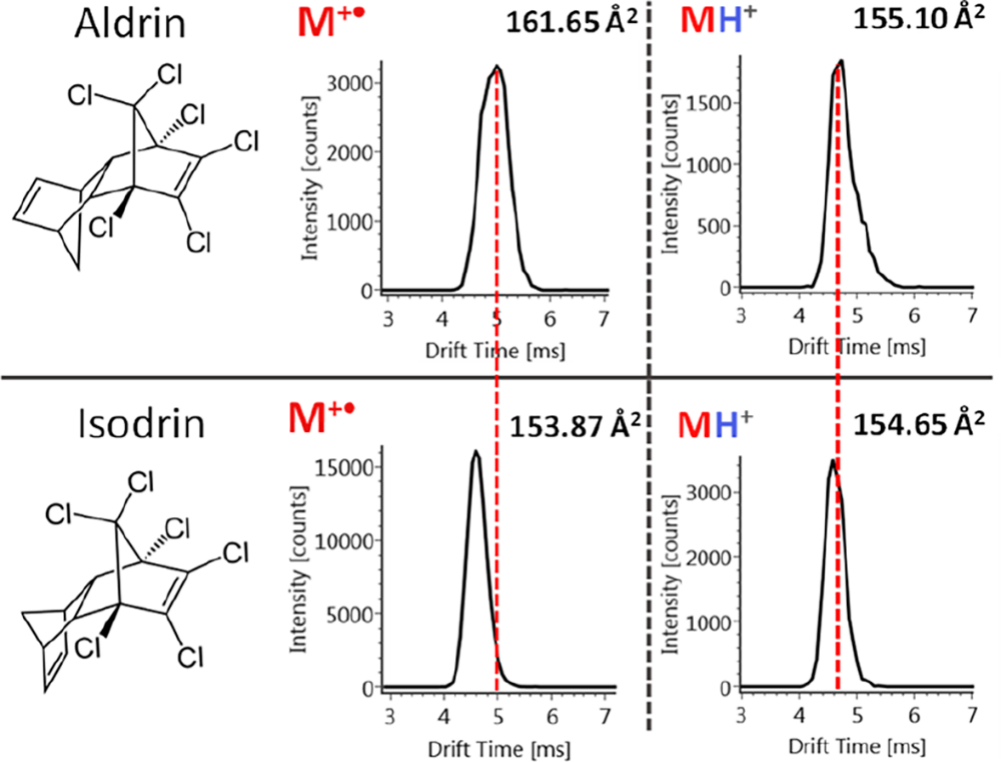
Mobilograms of the regioisomers aldrin (above) and isodrin (below) in charge transfer conditions (left) and proton transfer conditions (right).

Another interesting example is the pair aldrin/isodrin (Figure 3) for which similar CCS are obtained for the [M + H]^+^ species (155.10 and 154.65 Å^2^ respectively). However, this situation changes for the M^+•^ ions, whose CCS values (161.65 and 153.87 Å^2^ respectively) are different enough to separate both isomers by means of the IMS instrument used in this work. For this reason, both compounds were included in the list of charge transfer conditions (Table S2). In total, 23% of the molecules studied showed different behavior in the mobility cell depending on the ion species formed ([M + H]^+^ or M^+•^); therefore, it is of great help, when dealing with isomers, measuring in both ionization configurations to observe if CCS differences could improve the isomer discrimination.

#### Robustness of CCS Values for GC-Amenable Compounds

The preservation of the molecular ion and/or protonated molecule and the information contained in the fragmentation spectra in MS^E^, is one the main reasons for the increasing popularity of APCI source in GC–HRMS. However, unlike GC-EI-MS, the lack of commercial libraries is one of the main limitations for APCI-GC–HRMS implementation in suspect screening. The introduction of IMS in HRMS instruments could be a game changer as CCS measurements, in case of using the same IMS technology and/or a suitable calibration system, are expected to be little affected by different instruments used, improving the comparability between IMS-HRMS spectra for the same ion regardless of the ionization source.

CCS values for 84 [M + H]^+^ species acquired by GC-APCI-TWIMS-HRMS were compared with the equivalent adducts obtained by LC-ESI-TWIMS-HRMS reported by Celma et al.^18^Figure S3 illustrates the good correlation between the CCS provided by both techniques, showing a high degree of similarity (ΔCCS < 1%) for 70 of these compounds. Only for cyanophos (marked in blue), the CCS variation was higher than 2%. Upon inspection of the HE fragmentation pattern, no obvious explanation could be determined for this difference. The general agreement of CCS values obtained on GC and LC instruments is an encouraging step toward the implementation of GC-APCI-TWIMS-HRMS for suspect screening analysis by making use of CCS LC-TWIMS-HRMS databases (provided the compounds are GC-amenable and ionizable in wet conditions). Thus, molecular indicators, such as CCS and fragmentation, provided by online databases for [M + H]^+^, could be used for annotation purposes in GC-APCI-HRMS analysis and facilitate compound identification during suspect (and even nontarget) analyses. Furthermore, as previously indicated, M^+•^ provides CCS values similar to [M + H]^+^ in the 77% of the cases, opening the possibility of deploying CCS generated in both ionization configurations. In some occasions, the fragmentation observed in HE spectra for M^+•^ by APCI is comparable to that obtained in EI, making possible the use of the available electron ionization libraries (i.e., NIST) for identification purposes in some specific cases.^45^

The comparability of CCS values for GC-amenable organic pollutants obtained in different IMS-based platforms (e.g., DTIMS or TWIMS) should be further investigated in order to explore the possibility of a common CCS database. Figure S4 shows the CCS values for molecular and protonated ions of different PAHs obtained by DTIMS and TWIMS.^32^ Average errors were 1.3% for M^+•^ and 1.5% for [M + H]^+^ ions, showing a deviation of up to 2% in 6 out of a total of 23 ions but without exceeding 3%. These results are in accordance with the correlation between DTIMS and TWIMS for [M + H]^+^ and [M + Na]^+^ reported by Hinnenkamp et al., who remarked on the possibility of using CCS to exclude unambiguously incorrect assignment during identification, although the comparability between both instruments is not always possible.^26^

### Illustrative Examples of the Application of GC-APCI-IMS-QTOF MS for Screening Purposes

An automated workflow for GC-APCI-TWIMS-QTOF MS target screening, using the developed database for 264 GC-amenable compounds, was applied to different sample matrices, including four surface water samples, a fish feed and different fruit and vegetables commodities. Quality control samples were also included in the batch of analysis, consisting of representative samples spiked with a mixture of 182 compounds, and were used for confirmation purposes at different confidence levels (more details in the Supporting Information).

The benefits of using IMS in screening are illustrated in the example shown in Section 3.2.2., related to the identification of pentachloronitrobenzene in fish feed. In such applications, independent fragmentation spectra could be obtained at HE by using the appropriate DT alignment with LE spectra for each precursor ion, M^+•^ and [M + H]^+^, a fact that could be used to double-check the confirmation of potential positives in problematic cases. In addition, IMS improved the quality of the LE spectra, where numerous coeluting compounds made the interpretation troublesome. Using IMS, both ions [M + H]^+^ and M^+•^ could be present as the base peak in their corresponding LE spectra, where most of the matrix ions were “removed” (Figure 2B).

The improvement in the identification can also be observed in the screening of pesticide residues in water and food samples. Figures S5–S7 are illustrative examples of positive findings: terbumeton in RW, metalaxyl in tomato and fludioxonil in orange, respectively. Figures S5A, S6A, and S7A show GC-IMS-APCI-QTOF MS narrow window-XICs (mass window 0.01 Da) in both the sample and QC. The benefits of using IMS DT alignment (±0.2 ms) are clearly observed in Figures S5C, S6C, and S7C, which show much cleaner spectra in comparison with the conventional HRMS spectra at LE (Figures S5B, S6B, and S7B), a fact that clearly increases the reliability of the analyte identification.

In the screening applied to 12 samples, up to 74 positives were found (Table S4). It is worth noting the relevance to include the CCS deviation into the identification criteria as an extra value to enhance the confidence in the identification. In several cases, the mass accuracy criterion (mass error < 5 ppm) was not accomplished for the fragment ion (marked in Table S4 as F1, F2, etc.). This occurred for fludioxinil in several fruit and vegetable samples; thiabendazol and mephosfolan in apple; thiabendazol, propiconazol, and fenarimol in cauliflower, where mass errors exceeded 5 ppm. However, in all these cases, CCS deviation was below 2%, which together with the RT deviation criterion provided high reliability to the identification of these pesticides. Those examples support the inclusion of CCS into the identification criteria an extra value for enhancing the confidence in the identification process.

## Conclusions

The present work offers the first input of a wide CCS database applied for wide-scope screening of GC-amenable micropollutants using GC-APCI-TWIMS-HRMS. The in-house library was applied within an automated target screening workflow to several complex matrices, showing the potential of IMS to provide cleaner spectra and the possibility of using CCS values as extra point for improving the confidence in the identification process. Both dry and wet ionization modes show the general agreement between the CCS values of the molecular ion and/or protonated molecule for the same compound, with ΔCCS lower than 2% in more than 75% of the studied compounds. The opportunity offered by IMS to provide DT-aligned spectra is useful to obtain the fragment spectrum for each species (M^+•^ and [M + H]^+^), cleaning the coeluting interferences in complex matrices, with the possibility to discriminate some isomeric species. An interesting example of the power of GC-APCI-TWIMS-HRMS systems is the pair of isomers aldrin/isodrin, whose separation was not achieved in wet conditions but was possible in dry conditions. It is worth noting the excellent correlation between GC-APCI-TWIMS-HRMS mobility data for [M + H]^+^ species and the equivalent adducts acquired by LC-ESI-TWIMS-HRMS. This opens the door to the possibility of using CCS and fragmentation data from LC-HRMS databases to help in the identification of GC-amenable compounds during wide-scope screening based on the use of GC-APCI-HRMS.

## Supporting Information Available

The complete library of GC-amenable compounds for both wet and dry ionization conditions acquired through this work; specific examples and comparisons to literature references (PDF)
