# Supplementary material for: Benefits of Ion Mobility Separation in GC-APCI-HRMS Screening: From the Construction of a CCS Library to the Application to Real-World Samples
Source: Anal Chem. 2022 Jun 13;94(25):9040–7. doi: 10.1021/acs.analchem.2c01118 (PMC9974067; doi:10.1021/acs.analchem.2c01118)
Supplement: Supplementary file 1 — ac2c01118_si_001.pdf [file ac2c01118_si_001.pdf]

Supplementary Material

Benefits of ion mobility separation in GC-APCI-HRMS screening: from the construction of a CCS library to the application to real-world samples.

David Izquierdo-Sandoval[1], David Fabregat-Safont[1], Leticia Lacalle-Bergeron[1], Juan Sancho[1], Félix Hernández[1], Tania Portoles[1]*

[1] *Environmental and Public Health Analytical Chemistry, Research Institute for Pesticides and Water (IUPA), University Jaume I, Av. Sos Baynat S/N, 12071, Castellón de la Plana, Spain.*

*Dr. Tania Portolés (ORCID: 0000-0002-8422-0822) E-mail. tportole@uji.es

---

TABLE OF CONTENTS:

Title page....................S-1, S-2

Experimental section....................S-3

Chemical and Materials....................S-3

Samples selected as case study....................S-3, S-4

Table S1. Library for GC-APCI-IMS-HRMS in proton transfer conditions, including the compound name, CCS value, RSD(%) of the CCS, the family to which it belongs, the molecular formula and the five most representative fragments in HE. The suffix (_F) refers to the ion being an in-source fragment. LRI_{exp} refers to experimental Linear Retention Index....................S-5 - S-10

Table S2. Library for GC-APCI-IMS-HRMS in charge transfer conditions, including the compound name, CCS value, RSD(%) of the CCS, the family to which it belongs, the molecular formula and the five most representative fragments in HE. The suffix (_F) refers to the ion being an in-source fragment. LRI_{exp} refers to experimental Linear Retention Index....................S-10 - S-14

Table S3. Compounds showing CCS deviations between both ionic species $[M+H]^+$ and $M^{+\bullet}$ greater than 2%. S-15

Table S4. Screening of multiclass pesticides in river water samples, fruits and vegetable....................S-16, S-17

Figure S1. Average CCS value (n=6) and Relative Standard Deviation (RSD) for all ionic species (above) and for 50 randomly selected ion species, the "H" refers to $[M+H]^+$ whereas the absence of it refers to $M^{+\bullet}$ (bellow). In both graphs, ion species are ordered by means of average CCS values on the x-axis....................S-18

Figure S2. Comparison of HRMS spectra for quintozene in analytical reference standard solution at 10 µg L^{-1} (A), DT aligned data in fish feed sample spiked at 10 µg L^{-1} (B) and non-DT aligned data of the same finding in the same

spiked fish feed sample (C). Low energy (LE) and high energy (HE) spectra are shown for both species $[M+H]^+$ and $M^{+\bullet}$ S-19

Figure S3. Regression plot of the CCS values for protonated molecules from GC-APCI and LC-ESI. The blue circle surrounds cyanophos. S-20

Figure S4. Comparison of CCS values (Å^2) obtained by different IMS technologies for PAHs. CCS^{DTIMS} were taken for the work of Zheng et al.[5] and CCS^{TWIMS} from the library of the current work. S-20

Figure S5. Identification of terbumeton in surface water. (A) Extracted-ion chromatogram (XIC) (mass window ± 0.01Da) showing a positive finding in surface water. (B) Conventional LE (top) and HE (bottom) mass spectra without IMS drift time alignment. (C) LE (top) and HE (bottom) mass spectra with IMS drift time alignment. S-21

Figure S6. Identification of metalaxyl in tomato. (A) XIC (mass window ± 0.01Da) showing a positive finding in tomato. (B) Conventional LE (top) and HE (bottom) mass spectra without IMS drift time alignment. (C) LE (top) and HE (bottom) mass spectra with IMS drift time alignment. S-22

Figure S7. Identification of fludioxonil in orange. (A) XIC (mass window ± 0.01Da) showing a positive finding in orange. (B) Conventional LE (top) and HE (bottom) mass spectra without IMS drift time alignment. (C) LE (top) and HE (bottom) mass spectra with IMS drift time alignment. S-23

References S-24

EXPERIMENTAL SECTION

Chemical and Materials.

A total of 264 reference standards, purchased from different vendors, including 18 PCBs, 14 brominated flame retardants (BFRs), 16 organophosphate flame retardants (OPFRs), 23 PAHs, 182 multi-class pesticides and 11 emerging pollutants included insect repellents, musks and UV-filters among others, were injected for the development of an in-house library for screening purposes in GC-APCI-IMS-QTOF. Reference standards of pesticides, PCBs and PAHs were purchased from Dr. Ehrenstorfer (Augsburg, Germany). PBDE standard mixtures were purchased from Chiron (Trondheim, Norway). Musk standards were purchased from LGC Standards (Barcelona, Spain), and UV filters and insect repellents from Sigma Aldrich (Madrid, Spain).
From solid reference standards, stock solutions (around 500 μg mL^{-1}) were prepared by dissolving the standard in acetone and stored in a freezer at −20 ◦C. Different mixtures of reference standards of up to 15 compounds were prepared in hexane considering the substance classes, our previous knowledge about chromatographic separation, and avoiding the presence of isobaric and isomeric compounds in the same mixture. Mixtures at 10 and 100 μg L^{-1} were injected in triplicate, and analysed in both charge-transfer and proton-transfer conditions in the APCI source. Acetone (residue analysis and hexane (ultra-trace quality) were purchased from Scharlab (Barcelona, Spain). HPLC-grade water was obtained by purifying demineralised water in a Milli-Q Gradient A10 (Milli-pore, Bedford, MA, USA).

Samples selected as case study

River water samples

Four surface water samples were collected in different sampling sites (one sample from each sampling site) placed in the lower section and the estuary of the Mijares river in Eastern Spain. The sampling points correspond to the sites 16, 17, 18 and 19 selected by Bijlsma, et al [1]. Samples were collected in polyethylene bottles, transported in refrigerated isothermal containers and stored in the dark at -20 ◦C until their analysis.

Sample extraction and pre-concentration was made by solid-phase extraction (SPE). The procedure applied was based on the method previously developed by our research group [1]. Briefly, a volume of 250 mL of centrifuged water sample was passed through an Oasis HLB cartridge (150 mg, 6cc, Waters), previously conditioned and equilibrated, and then, the analytes were eluted with 5 mL of methanol. The extract was evaporated to dryness and reconstituted with 250 µL of hexane. Quality control (QC) were included, consisted of three aliquots of a mixture of the four surface water samples in equal parts each one fortified at three concentration levels with 182 compounds (0.01, 0.1 and 1 µg L^{-1}) before the extraction.

Fish feed sample

Briefly, 1 g of fish feed sample was weighted into a 15 mL Falcon tube and 2 mL of acetonitrile were added. After shaking for 30 s, 0.8 g of anhydrous magnesium sulfate were added and immediately shaken vigorously for 30 s. The tubes were centrifuged at 1893 rcf·g for 5 min and the upper layer of the extract was transferred to a 2 mL eppendorf tube and stored for at least two hours in a freezer to precipitate proteins and fix lipids to the tube walls (freezing cleanup). Then, 1 mL of the extract was carefully transferred to the cleanup QuEChERS vial (50 mg of PSA +150 mg of anhydrous magnesium sulfate +50 mg of C18), and it was vigorously shaken for 30 s and centrifuged at 12,557 rcf·g for 5 min. Then, 50 µL of the final acetonitrile extract was diluted with 300 µL of acetone and 650 µL of hexane in order to make the solution miscible (20-fold dilution factor). The extract was spiked at 10 and 50 µg L^{-1} with 182 compounds for QC. More details in Portolés et al. [2].

Fruits and vegetables commodities

An acetate-buffered version of the QuEChERS method with some modifications was followed as the extraction procedure, as was applied in a previous study in our laboratory [3]. Briefly, 10 g of chopped and homogenized sample were weighed in a 50 mL Falcon conical tube and 10 mL 1% acetic acid in acetonitrile (v/v) were added. After shaking for 30 s, 1 g of anhydrous sodium acetate and 4 g of anhydrous magnesium sulfate were added and immediately shaken vigorously for 1 min. The tubes were centrifuged at 1893 rcf·g for 5 min and then 2 aliquots of the supernatant of 1 mL each, were transferred into separate d-SPE tubes containing 50 mg PSA, 150 mg anhydrous magnesium sulfate and 150 mg C18 for clean-up purposes and vortexed for 30 s. After centrifugation at 1150 rfc·g for 2 min, 500 µL of supernatant from each of the tubes were combined into an graduated evaporation tube, where it was evaporated under a gentle nitrogen stream at 50 ◦C to approximately 250 µL. The final volume was adjusted to 300 µL resulting in a final extract of 3.33 g matrix equivalent/mL. Apple and orange samples were fortified by addition of a mix solution of 182 compounds to 10 g portions of the homogenized materials at three levels (0.002, 0.01 and 0.05 mg kg^{-1}), representing "high water content" and "high acid and high water content" matrices respectively as it is defined in SANTE/12682/2019 guideline [4]. These samples were used as Quality Control.

Table S1. Library for GC-APCI-IMS-HRMS in <u>proton transfer conditions</u>, including the compound name, CCS value, RSD(%) of the CCS, the family to which it belongs, the molecular formula and the five most representative fragments in HE. The suffix (_F) refers to the ion being an in-source fragment. LRI_{exp} refers to experimental Linear Retention Index.

Compound	CCS (Å^2)	RSD (%)	Family	Formula	LRI_{exp}	F1	F2	F3	F4	F5
DEET	143.01	0.08	Insect repellent	C12H17NO	1562	119.04957	91.05503	72.04510	172.11329	
ambrettolide	160.94	0.10	Musk	C16H28O2	1905	235.20536	217.19500	95.08633	81.07051	119.08572
cashmeran	148.04	0.08	Musk	C14H22O	1488	137.09631	189.16389	177.12744	133.10133	151.11179
musk ketone	170.47	0.08	Musk	C14H18N2O5	1931	232.13322	148.07529	174.09101	217.10900	190.08559
musk xylene	168.06	0.08	Musk	C12H15N3O6	1830	147.06578	118.06498	235.10658	264.09716	158.09629
muskone	161.17	0.07	Musk	C16H30O	1826	213.16347	171.11659	95.08613	109.10147	183.11660
tonalide	168.29	0.10	Musk	C18H26O	1836	175.11156	119.08545	133.010115	147.08020	161.09589
traseolide	168.93	0.09	Musk	C18H26O	1826	189.12786	147.08033	173.13324	129.06971	215.17994
4,4'dichlorobenzophenone	150.42	0.12	Pesticides	C13H8Cl2O	1960	139.99469	110.99991	75.02383		
alpha endosulfan	166.32	0.19	Pesticides	C9H6Cl6O3S	2107	322.85173	286.87524	250.89824	216.93717	204.93704
atrazine	148.98	0.44	Pesticides	C8H14ClN5	1740	174.05378	138.07749	132.03235	104.00160	96.05601
atrazine desethyl	138.64	0.24	Pesticides	C6H10ClN5	1655	146.02285	104.00167	68.02552	110.04696	83.06169
atrazinedesisopropyl	132.00	0.06	Pesticides	C5H8ClN5	1642	132.03257	68.02539	104.00200	146.02302	
azaconazole	160.93	0.21	Pesticides	C12H11Cl2N3O2	2173	158.97643	230.99778	256.00413	82.04022	
azinphos ethyl	174.71	0.49	Pesticides	C12H16N3O3PS2	2595	260.97930	232.94040	137.00552	132.04440	96.95132
azinphos methyl_F	117.28	0.10	Pesticides	C8H5NO	2513	105.03409	104.05045	77.03991		
azoxystrobin	194.46	0.10	Pesticides	C22H17N3O5	3062	372.09750	344.10260			
beta endosulfan	164.93	0.22	Pesticides	C9H6Cl6O3S	2229	322.85173	286.87524	250.89824	216.93717	204.93704
bixafen	181.57	0.20	Pesticides	C18H12Cl2F3N3O	2835	159.03628	265.99328	374.02596	394.03127	338.48500
bromophos ethyl	166.26	0.08	Pesticides	C10H12BrCl2O3PS	2066	338.82264	161.96340	177.94030	96.95101	258.85520
bromophos methyl	157.19	0.07	Pesticides	C8H8BrCl2O3PS	1963	334.82782	124.98226	109.00539	212.86894	240.86353
bromuconazole I	168.34	0.16	Pesticides	C13H12BrCl2N3O	2412	158.97572	172.95503	213.99321	192.0336	296.03536
bromuconazole II	166.65	0.21	Pesticides	C13H12BrCl2N3O	2420	158.97572	172.95503	208.99133	192.03341	
buprofezin	174.19	0.09	Pesticides	C16H23N3OS	2187	250.10019	201.10568	145.04300	134.06022	106.06572
cadusafos	157.61	0.09	Pesticides	C10H23O2PS2	1676	130.9365	158.96994	96.95151		
captan_F	145.34	0.26	Pesticides	C9H7Cl2NO2S	2006	235.97040	179.94391	171.99834	155.90719	147.97187

Compound	CCS (Å^2)	RSD (%)	Family	Formula	LRI_{exp}	F1	F2	F3	F4	F5
carbofuran	147.99	0.09	Pesticides	C12H15NO3	1736	123.04452	165.09122			
carbosulfan	190.66	0.13	Pesticides	C20H32N2O3S	2429	160.11536	118.06856	128.14335	104.05330	76.02240
chinomethionate	143.66	0.16	Pesticides	C10H6N2OS2	2044	175.03176	207.00362	104.04921	121.01019	116.04916
chlordecone	165.22	0.12	Pesticides	C10Cl10O	2280	236.84065	218.87454	419.73837	454.70763	354.77640
chlorfenapyr	177.74	0.17	Pesticides	C15H11BrClF3N2O	2200	271.02461	327.05091	378.96500	362.93316	282.01697
chlorfenson	161.35	0.14	Pesticides	C12H8Cl2O3S	2143	174.96160	126.99482	111.00014		
chlorfenvinfos	167.00	0.04	Pesticides	C12H14Cl3O4P	2007	204.93763	169.96849	155.04698	127.0157	98.98499
chlorothalonil	150.03	0.49	Pesticides	C8Cl4N2	1781	229.91992	228.91221	132.97165	167.94034	193.94357
chlorpyrifos ethyl	162.97	0.16	Pesticides	C9H11Cl3NO3PS	1944	321.90236	293.87094	275.86063	197.9274	124.98218
chlorpyrifos methyl	153.47	0.07	Pesticides	C7H7Cl3NO3PS	1870	289.87606	124.98226			
clodinafop-propargyl	179.85	0.12	Pesticides	C17H13ClFNO4	2336	266.03792	238.00648	176.04995	129.98470	
coumaphos	179.62	0.07	Pesticides	C14H16ClO5PS	2692	334.99013	306.95889	288.94835	226.99261	211.01546
cyanazine	155.30	0.15	Pesticides	C9H13ClN6	1954	214.08539	205.11981	132.03621	104.00176	83.06161
cyanophos	154.25	0.09	Pesticides	C9H10NO3PS	1769	211.92540	124.98229	109.00534	134.00588	150.03674
cyfluthrin I	195.84	0.15	Pesticides	C22H18Cl2FNO3	2753	226.06573	191.00154	163.00750	127.03120	91.05497
cyfluthrin II	200.53	0.24	Pesticides	C22H18Cl2FNO3	2768	226.06573	191.00154	163.00750	127.03120	91.05497
cyfluthrin III	200.53	0.24	Pesticides	C22H18Cl2FNO3	2773	226.06573	191.00154	163.00750	127.03120	91.05497
cyfluthrin IV	200.44	0.24	Pesticides	C22H18Cl2FNO3	2782	226.06573	191.00154	163.00750	127.03120	91.05497
cypermethrin I	195.52	0.13	Pesticides	C22H19Cl2NO3	2794	191.00154	163.00750	127.03120	91.05497	
cypermethrin II	199.97	0.35	Pesticides	C22H19Cl2NO3	2811	191.00154	163.00750	127.03120	91.05497	
cypermethrin III	199.97	0.35	Pesticides	C22H19Cl2NO3	2816	191.00154	163.00750	127.03120	91.05497	
cypermethrin IV	200.33	0.18	Pesticides	C22H19Cl2NO3	2824	191.00154	163.00750	127.03120	91.05497	
cyproconazole	167.55	0.02	Pesticides	C15H18ClN3O	2188	125.01545	70.04032			
cyprodinil	153.51	0.15	Pesticides	C14H15N3	1987	210.10252	184.08674	167.0724	144.08053	119.06029
deltamethrin	197.63	0.52	Pesticides	C22H19Br2NO3	3037	278.90143	199.98364	171.98813	121.06502	
diazinon	169.05	0.20	Pesticides	C12H21N2O3PS	1793	277.07634	266.99865	249.04494	169.07885	153.10168
dichlofenthion	158.17	0.07	Pesticides	C10H13Cl2O3PS	1854	258.91502	240.90461	178.94836	142.99009	96.95126
dichloran	132.71	0.11	Pesticides	C6H4Cl2N2O2	1711	189.96190	159.97116	123.99466	175.96606	147.97162
dichlorvos	133.63	0.19	Pesticides	C4H7Cl2O4P	1239	127.01581	109.00558	94.99080	78.99565	
dicrotophos	147.62	0.18	Pesticides	C8H16NO5P	1659	127.01513	112.07627	72.04470		

Compound	CCS (Å^2)	RSD (%)	Family	Formula	LRI_{exp}	F1	F2	F3	F4	F5
dieldrin	157.92	0.31	Pesticides	C12H8Cl6O	2163	324.89091	288.91427	289.92136	260.86009	253.94512
difenoconazole	197.92	0.05	Pesticides	C19H17Cl2N3O3	2974	251.00249	337.0393	188.03846	82.04000	
dimethachlor	151.73	0.17	Pesticides	C13H18ClNO2	1852	224.08389	148.11221	132.08099	105.07076	174.09192
dimethoate	140.17	0.09	Pesticides	C5H12NO3PS2	1709	124.98206				
diphenylamine	136.33	0.07	Pesticides	C12H11N	1619	152.06147	93.05766	92.05002		
endosulfan ether	152.53	0.20	Pesticides	C9H6Cl6O	1834	286.87405	274.87524	250.89860	216.93710	204.93649
endrin	163.84	0.23	Pesticides	C12H8Cl6O	2194	314.90631	306.92433	278.92949	243.96029	242.95305
EPN	168.87	0.11	Pesticides	C14H14NO4PS	2436	156.98723	296.01429	262.02654	141.01012	278.00434
epoxiconazole	171.31	0.15	Pesticides	C17H13ClFN3O	2381	121.04412	149.03981	101.03778		
ethalfluralin	165.31	0.16	Pesticides	C13H14F3N3O4	1648	232.03260	276.05917	202.03452	186.03967	300.09556
ethion	175.39	0.06	Pesticides	C9H22O4P2S4	2263	199.00117	170.96971	142.93842	124.98226	96.95128
ethoxyquin	154.63	0.14	Pesticides	C14H19NO	1730	190.12215	148.07511	176.10579	162.09089	158.09597
famphur	169.47	0.07	Pesticides	C10H16NO5PS2	2295	217.00861	280.97009	124.98265	93.01101	184.98231
fenarimol	174.30	0.24	Pesticides	C17H12Cl2N2O	2585	304.02967	268.05259	259.00785	189.07033	138.99494
fenhexamid	165.56	0.22	Pesticides	C14H17Cl2NO2	2328	97.10228	143.01353	176.97466	266.09469	
fenitrothion	156.09	0.22	Pesticides	C9H12NO5PS	1923	245.99831	213.97216	200.00497	168.98651	124.98207
fenoxycarb	179.77	0.07	Pesticides	C17H19NO4	2469	256.09638	88.03979	116.07064	70.03030	213.05435
fenpropimorph	174.70	0.18	Pesticides	C20H33NO	1952	147.11683	119.08575	130.12188		
fenthion	156.70	0.12	Pesticides	C10H15O3PS2	1949	247.00119	169.01378	153.03666	138.04933	124.98202
fipronil	180.21	0.12	Pesticides	C12H4Cl2F6N4OS	1992	367.94952	254.96991	314.97083	289.97500	221.00841
flumethrin	218.53	0.34	Pesticides	C28H22Cl2FNO3	3086					
folpet_F	141.78	0.16	Pesticides	C9H3Cl2NO2S	2011	116.90620				
ioxynil-octanoate	183.55	0.10	Pesticides	C15H17I2NO2	2578	371.83809	244.93310	127.11205		
iprodione	178.96	0.13	Pesticides	C13H13Cl2N3O3	2437	244.98814	216.99341	187.96661	173.98713	161.98741
iprovalicarb	186.10	0.14	Pesticides	C18H28N2O3	2174	119.08570	134.09610	91.05460	98.05923	
lambda cyhalothrin I	199.44	0.05	Pesticides	C23H19ClF3NO3	2558	225.02889	197.03386	181.06469	161.05728	141.05115
lambda cyhalothrin II	199.50	0.09	Pesticides	C23H19ClF3NO3	2591	225.02889	197.03386	181.06469	161.05728	141.05115
leptophos	170.32	0.08	Pesticides	C13H10BrCl2O2PS	2511	171.00257	380.84858	155.02552	138.97645	124.03389
malathion	165.89	0.16	Pesticides	C10H19O6PS2	1935	285.00175	127.03925	124.98221	99.00808	
mepanipyrim	153.05	0.09	Pesticides	C14H13N3	2120	182.08385	168.06383	131.06088	106.06565	143.0615

Compound	CCS (Å²)	RSD (%)	Family	Formula	LRI_{exp}	F1	F2	F3	F4	F5
mephosfolan	152.76	0.02	Pesticides	C8H16NO3PS2	1993	139.95583	167.98752	196.01930		
metalaxyl	160.53	0.13	Pesticides	C15H21NO4	1902	192.13842	160.11216	220.13347	148.11213	248.12802
metconazole	167.70	0.17	Pesticides	C17H22ClN3O	2467	125.01531	177.04673	233.10927	302.14172	70.04011
methidathion	151.99	0.27	Pesticides	C6H11N2O4PS3	2054	286.99215	145.00649			
methiocarb	152.26	0.06	Pesticides	C11H15NO2S	1919	169.06785	153.03684	121.06501	107.04951	91.05492
methoxychlor_F	139.04	0.21	Pesticides	C9H7Cl3O	2455	201.99465	186.9711	167.02564	158.97617	132.05683
metolachlor	159.29	0.07	Pesticides	C15H22ClNO2	1939	252.11518	176.14328	160.11182	146.09634	134.09626
metrafenone	181.75	0.10	Pesticides	C19H21BrO5	2595	209.08085	228.96841	166.06194	194.05732	
metribuzin	147.10	0.15	Pesticides	C8H14N4OS	1872	187.10103	171.05877	145.054	114.99669	89.01735
molinate	138.41	0.23	Pesticides	C9H17NOS	1536	126.09151	98.09698	83.08638	55.05520	160.11556
omethoate	135.20	0.08	Pesticides	C5H12NO4PS	1583	124.98238	109.00588	154.99284	182.9876	196.01964
oxadixyl	157.69	0.21	Pesticides	C14H18N2O4	2251	219.11293	191.09378	133.08780	132.08093	
parathion ethyl	159.64	0.08	Pesticides	C10H14NO5PS	1954	235.97786	217.96721	189.98479	171.97424	156.01057
parathion methyl	150.10	0.14	Pesticides	C8H10NO5PS	1882	231.98286	199.95682	185.99004	154.97146	109.00547
pendimethalin	159.89	0.17	Pesticides	C13H19N3O4	1986	264.13449	236.10299	230.12883	212.0667	201.13835
permethrin I_F	185.49	0.08	Pesticides	C21H19ClO3	2675	337.09854	319.13264	301.12216	273.1276	255.05729
permethrin II_F	187.37	0.11	Pesticides	C21H19ClO3	2693	319.13241	279.09879	275.14274	261.09083	255.05773
phorate	149.05	0.26	Pesticides	C7H17O2PS3	1687	170.96958	142.93850	124.98207	96.95130	75.02709
phosmet	158.56	0.15	Pesticides	C11H12NO4PS2	2434	160.03923	133.02847			
phosphamidon	156.16	0.13	Pesticides	C10H19ClNO5P	1853	226.98729	127.0156	174.06796	100.07610	109.00612
pirimicarb	153.29	0.11	Pesticides	C11H18N4O2	1837	182.12872	166.09761	150.10267	137.07102	72.04524
pirimiphos methyl	165.36	0.07	Pesticides	C11H20N3O3PS	1924	164.11820	151.02665	136.08693	124.98232	108.05598
procymidone	159.64	0.08	Pesticides	C13H11Cl2NO2	2033	256.02909	238.01871	228.03435	212.00276	185.98717
profenofos	166.61	0.13	Pesticides	C11H15BrClO3PS	2152	304.86197	187.96964	266.88772	128.00300	143.97956
propachlor	143.12	0.05	Pesticides	C11H14ClNO	1594	170.03715	106.06630	152.02651	134.06087	94.06632
propazine	155.64	0.21	Pesticides	C9H16ClN5	1744	188.06975	146.02294	104.00143	68.02346	
propiconazole	176.88	0.05	Pesticides	C15H17Cl2N3O2	2326	158.97628	256.00408	186.97158	172.95563	69.07073
propoxur_F	130.31	0.12	Pesticides	C8H9NO3	1606	111.04393	93.03390	65.03940		
propyzamide	157.63	0.18	Pesticides	C12H11Cl2NO	1779	189.98201	172.95549			
prosulfocarb	158.24	0.06	Pesticides	C14H21NOS	1907	91.05489	128.10706			

Compound	CCS (Å^2)	RSD (%)	Family	Formula	LRI_{exp}	F1	F2	F3	F4	F5
pyriproxyfen	183.91	0.10	Pesticides	C20H19NO3	2544	227.10671	185.05975	96.04509	78.03474	
quinalphos	162.45	0.05	Pesticides	C12H15N2O3PS	2025	271.03011	242.99874	224.98827	156.06786	147.05543
quintozene	146.24	0.35	Pesticides	C6Cl5NO2	1723	248.84089	264.84410	213.87178	176.90561	
simazine	142.39	0.22	Pesticides	C7H12ClN5	1738	174.05412	166.10864	132.03268	124.08702	104.00157
tau-fluvalinate I	218.12	0.07	Pesticides	C26H22ClF3N2O3	2940	250.06023	208.07570	181.06483		
tau-fluvalinate II	217.44	0.15	Pesticides	C26H22ClF3N2O3	2953	250.06023	208.07570	181.06483		
tebuconazole	166.25	0.03	Pesticides	C16H22ClN3O	2349	290.14194	125.01549	165.04650	103.05385	70.04010
terbufos_F	136.16	0.21	Pesticides	C5H11O2PS3	1763	128.92271	174.91020	156.95041	184.98643	96.95120
tefluthrin	176.40	0.07	Pesticides	C17H14ClF7O2	1828	325.04634	177.03218	127.03543		
terbumeton	156.15	0.05	Pesticides	C10H19N5O	1760	170.10340	142.07230	100.05086	114.06611	128.08190
terbumetondesethyl	145.32	0.23	Pesticides	C8H15N5O	1652	142.07282	100.05151	86.03614		
terbuthylazine	154.17	0.08	Pesticides	C9H16ClN5	1774	174.05414	146.02290	138.07747	132.03244	104.00165
terbuthylazine desethyl	143.80	0.12	Pesticides	C7H12ClN5	1665	146.02315	104.00187	68.02547	79.00682	
terbutryn	160.25	0.14	Pesticides	C10H19N5S	1919	186.08063	158.04943	170.04913	138.07732	116.02786
tetradifon	165.97	0.33	Pesticides	C12H6Cl4O2S	2504	242.88364	194.91667	178.92168	174.96148	158.96665
thiabendazole	136.58	0.01	Pesticides	C10H7N3S	1988	175.03154	131.06003			
tolclofos-methyl	150.20	0.11	Pesticides	C9H11Cl2O3PS	1875	268.93488	174.97074	124.98183	253.91120	109.00707
tolyfluanid_F	139.44	0.14	Pesticides	C8H6Cl2FNS	1990	213.06956	137.02959	91.05505		
triadimefon	172.25	0.07	Pesticides	C14H16ClN3O2	1953	197.07282	225.06761	141.01038	155.02605	111.00052
triflumizole	174.71	0.19	Pesticides	C15H15ClF3N3O	2046	355.06924	346.09181	326.96558	278.05500	266.02627
trifluralin	168.43	0.18	Pesticides	C13H16F3N3O4	1673	290.07464	260.06415	248.02774	232.03287	214.07138
vinclozolin	162.10	0.18	Pesticides	C12H9Cl2NO3	1881	242.01333	214.00074	178.04184	171.97149	164.02603
T2IPPP	204.49	0.14	OPFRs	C27H33O4P	2723	411.17195	369.12477	327.07818	293.09291	234.0395
T35DMPP	203.66	0.05	OPFRs	C24H27O4P	2857	307.10912	289.09886	271.08815	194.10859	179.08541
TBEP	198.71	0.08	OPFRs	C18H39O7P	2392	343.18897	299.16203	243.09889	225.08866	199.07320
TBPP	239.81	0.12	OPFRs	C30H39O4P	3349	439.20317	383.14054			
TCEP	149.21	0.07	OPFRs	C6H12Cl3O4P	1748	222.96903	186.99274	160.97639	125.00000	98.98458
TCPP	160.42	0.18	OPFRs	C9H18Cl3O4P	1778	251.0008	174.99235	139.01579	98.98458	
TDBPP	188.91	0.09	OPFRs	C9H15Br6O4P	3005	498.71573	298.85016	200.87328	136.99991	118.94958
TDCPP	170.49	0.02	OPFRs	C9H15Cl6O4P	2292	320.91926	208.95312	284.94313	172.97640	137.00009

Compound	CCS (Å²)	RSD (%)	Family	Formula	LRI_{exp}	F1	F2	F3	F4	F5
TEHP	221.61	0.09	OPFRs	C24H51O4P	2443	352.22481	240.09958	225.09011	127.97457	98.98471
TMTP	187.69	0.08	OPFRs	C21H21O4P	2617	279.07788	261.06731	243.05677	196.08793	165.06981
TNBP	167.05	0.12	OPFRs	C12H27O4P	1637	211.10918	184.03677	155.04686	98.98471	
TOTP	180.58	0.06	OPFRs	C21H21O4P	2543	277.06208	165.07009	179.08541	261.06743	243.05692
TPP	170.49	0.09	OPFRs	C18H15O4P	2366	309.06842	251.04673	233.03626	215.02574	152.06193
TPRP	149.05	0.07	OPFRs	C9H21O4P	1372	139.01594	98.98466	80.97441		
TPTP	188.78	0.09	OPFRs	C21H21O4P	2709	279.07852	261.06750	243.06750	198.10334	166.07719
octocrylene	193.47	0.08	UV-filters	C24H27NO2	2623	232.07594	204.08072	250.08632	177.06910	105.03433

Table S2. Library for GC-APCI-IMS-HRMS in charge transfer conditions, including the compound name, CCS value, RSD(%) of the CCS, the family to which it belongs, the molecular formula and the five most representative fragments in HE. The suffix (_F) refers to the ion being an in-source fragment. LRI_{exp} refers to experimental Linear Retention Index.

Compound	CCS (Å²)	RSD (%)	Family	Formula	LRI_{exp}	F1	F2	F3	F4	F5
BDE 28	156.47	0.30	BFRs	C12H7Br3O	2245	327.89151	298.88925	245.96748	247.96555	168.05620
BDE 47	162.52	0.14	BFRs	C12H6Br4O	2492	405.80205	325.87697	245.96732	216.96507	296.87534
BDE 66	163.88	0.07	BFRs	C12H6Br4O	2537	405.80119	325.87660	245.96758	216.96469	234.85795
BDE 85	168.69	0.18	BFRs	C12H5Br5O	2865	485.71048	403.78709	325.87608	296.87384	250.85322
BDE 99	170.49	0.37	BFRs	C12H5Br5O	2757	485.71084	403.78753	325.87627	250.85332	296.87383
BDE 100	168.11	0.07	BFRs	C12H5Br5O	2690	485.70749	483.70984	403.78736	325.87656	296.87408
BDE 153	177.57	0.13	BFRs	C12H4Br6O	3015	563.61901	483.69607	403.78765	330.76248	
BDE 154	175.19	0.17	BFRs	C12H4Br6O	2921	563.61974	483.69588	405.78469	330.76137	
BDE 183	180.63	0.08	BFRs	C12H3Br7O	3274	643.52930	561.60649	483.69606	408.67247	
BDE 184	177.00	0.11	BFRs	C12H3Br7O	3220	643.52968	561.60652	483.69508	408.67240	
BDE 191	182.75	0.09	BFRs	C12H3Br7O	3349	643.52839	561.60655	483.69508	408.67240	
BDE 196	186.85	0.15	BFRs	C12H2Br8O	3634	721.43697	641.51371	561.60566	408.67240	
BDE 197	182.94	0.15	BFRs	C12H2Br8O	3564	721.44115	641.51458	563.60348	408.67374	
BTBPE	182.34	0.18	BFRs	C14H8Br6O2	3353	356.79509	277.87657	330.78061	249.88212	196.95969
ehmc	183.43	0.02	UV-filters	C18H26O3	2316	178.06234	161.05972	132.05707	117.03376	77.03911
galaxolide_F	161.01	0.24	Musk	C17H22O	1840	213.16364	171.11590	143.08472	198.13981	129.06891

Compound	CCS (Å^2)	RSD (%)	Family	Formula	LRI_{exp}	F1	F2	F3	F4	F5
5-methylchrysene	149.18	0.08	PAHs	C19H14	2573	215.08560	228.09324	202.07749	165.07019	
acenaphthene	127.67	0.14	PAHs	C12H10	1449	127.05434	115.05433	141.06998	77.03884	102.04668
acenaphthylene	125.25	0.21	PAHs	C12H8	1449	139.05398	126.04607	102.04620	78.04592	89.03842
anthracene	131.03	0.06	PAHs	C14H10	1785	165.07004	152.06198	128.06211	115.05462	139.05469
benzo(a)anthracene	144.42	0.12	PAHs	C18H12	2430	215.08580	202.07793	189.06970	178.07755	
benzo(a)pyrene	148.43	0.10	PAHs	C20H12	2821	239.08560	226.07726	202.07763	189.07035	
benzo(b)fluoranthene	149.83	0.04	PAHs	C20H12	2736	226.07777	239.08571	202.07773	215.08502	
benzo(c)fluorene	141.98	0.07	PAHs	C17H12	2213	202.07769	189.06978	165.07008		
benzo(ghl)perylene	153.08	0.13	PAHs	C22H12	3191	263.08600	250.07825			
benzo(k)fluoranthene	150.80	0.03	PAHs	C20H12	2745	239.08568	226.07780	202.07785	213.07063	189.07100
benzo[j]fluoranthene	149.99	0.11	PAHs	C20H12	2740	226.07744	239.08514	202.07707		
chrysene	143.58	0.16	PAHs	C18H12	2440	202.07750	192.98082	215.08585	189.07001	152.06160
dibenzo(a,h)anthracene	158.92	0.09	PAHs	C22H14	3146					
dibenzo[a,e]pyrene	160.98	0.03	PAHs	C24H14	3569					
dibenzo[a,h]pyrene	161.28	0.12	PAHs	C24H14	3597					
dibenzo[a,i]pyrene	161.12	0.16	PAHs	C24H14	3600					
dibenzo[a,l]pyrene	163.27	0.18	PAHs	C24H14	3496	289.10142	276.09330			
fluoranthene	136.79	0.08	PAHs	C16H10	1996	189.07017	152.06220	176.06235	163.05452	128.06231
fluorene	129.33	0.17	PAHs	C13H10	1580	152.06220	139.05433	115.04560	89.03939	128.06234
indeno(123cd)pyrene	155.22	0.10	PAHs	C22H12	3129	263.08588	250.07724	239.08427		
naphthalene	120.30	0.24	PAHs	C10H8	1195	102.04582	78.04583			
phenanthrene	130.74	0.15	PAHs	C14H10	1774	152.06148	165.06940	128.06160	139.05402	115.05398
pyrene	136.00	0.07	PAHs	C16H10	2097	189.06993	176.06152	152.06134	163.05408	
PCB 28	144.79	0.28	PCBs	C12H7Cl3	1867	186.02336	220.99200	151.05419		
PCB 52	151.98	0.19	PCBs	C12H6Cl4	1923	219.98414	254.95303	185.01489	150.04617	
PCB 77	152.09	0.24	PCBs	C12H6Cl4	2160	219.98414	232.02420	257.95629	185.01479	150.04619
PCB 81	152.65	0.20	PCBs	C12H6Cl4	2181	219.98421	233.97835	255.96095	185.01481	150.04661
PCB 101	157.62	0.27	PCBs	C12H5Cl5	2106	253.94549	290.91151	218.97687	184.00687	
PCB 105	157.88	0.17	PCBs	C12H5Cl5	2225	253.94573	290.91174	267.98286	218.97635	184.00748
PCB 114	156.35	0.15	PCBs	C12H5Cl5	2252	253.94511	267.98268	290.91150	218.97588	184.00715

Compound	CCS (Å^2)	RSD (%)	Family	Formula	LRI_{exp}	F1	F2	F3	F4	F5
PCB 118	158.16	0.18	PCBs	C12H5Cl5	2233	253.94569	290.91164	218.96610	184.00708	
PCB 123	156.79	0.14	PCBs	C12H5Cl5	2284	253.94535	265.98512	292.90979	218.97608	184.00797
PCB 126	158.96	0.10	PCBs	C12H5Cl5	2357	253.94537	265.98590	291.91805	218.97662	184.00787
PCB 138	162.22	0.26	PCBs	C12H4Cl6	2334	289.90365	324.87264	254.93508	217.96920	
PCB 153	163.51	0.21	PCBs	C12H4Cl6	2279	289.90356	324.87222	253.64548	217.96814	
PCB 156	164.54	0.19	PCBs	C12H4Cl6	2398	289.90355	301.94351	324.87233	253.94491	
PCB 157	163.07	0.13	PCBs	C12H4Cl6	2445	289.90351	301.94400	324.87226	254.93536	
PCB 167	162.83	0.12	PCBs	C12H4Cl6	2455	289.90356	301.94355	324.87250	253.94511	217.96802
PCB 169	165.60	0.13	PCBs	C12H4Cl6	2531	289.90328	301.94327	325.87802	255.94005	
PCB 180	168.12	0.25	PCBs	C12H3Cl7	2482	323.86454	358.83354	289.90218		
PCB 189	169.25	0.13	PCBs	C12H3Cl7	2612	323.86474	335.90461	358.83449	289.90460	
2-phenylphenol	130.74	0.10	Pesticides	C12H10O	1514	152.06191	141.06998	141.06998	115.05454	77.03938
acequinocyl_F	196.61	0.14	Pesticides	C22H30O3	2854	323.20017	215.03339	203.10762		
alachlor	155.59	0.14	Pesticides	C14H20ClNO2	1887	188.10659	172.11187	160.11178	131.07217	
aldrin	161.65	0.94	Pesticides	C12H8Cl6	1936	247.85165	213.89023			
bifenthrin_F	137.00	0.24	Pesticides	C14H12	2464	166.07640				
bromopropylate_F	154.65	0.49	Pesticides	C13H8Br2O	2451	184.94194	154.94921			
captafol	158.09	0.07	Pesticides	C10H9Cl4NO2S	2376	160.87801	275.96480	311.94104	148.03926	107.04961
carbophenothion	169.85	0.07	Pesticides	C11H16ClO2PS3	2314	156.98731	142.93870	170.96967	199.00132	124.98222
carfentrazone ethyl	183.89	0.13	Pesticides	C15H14Cl2F3N3O3	2321	312.05904	340.09041	330.02511	976.06690	240.99777
chlorpropham	145.61	0.61	Pesticides	C10H12ClNO2	1658	171.00809	127.01836			
DDD_F	144.47	0.29	Pesticides	C13H8Cl2	2253	199.03052	165.06924			
DDE	161.83	0.06	Pesticides	C14H8Cl4	2166	280.96878	245.99920	210.02300		
DDT_F	144.84	0.11	Pesticides	C13H8Cl2	2335	199.03051	164.06122			
deltamethrin	194.82	0.20	Pesticides	C22H19Br2NO3	3037	397.04220	344.12784	278.90061	250.90668	208.07503
dicofol_F	150.52	0.01	Pesticides	C13H8Cl2O	2466	138.99394	110.99939			
difenoconazole_F	173.40	0.03	Pesticides	C16H12Cl2O3	2995	264.98190	202.01794	173.01531	209.00910	
diflufenican	183.86	0.07	Pesticides	C19H11F5N2O2	2389	266.04230	246.03589	238.04706	375.07444	
dioxathion_F	147.24	0.13	Pesticides	C8H15O4PS2	1758	140.97660	169.00802	197.03938	96.95089	242.99170
endosulfan sulfate	156.41	0.34	Pesticides	C9H6Cl6O4S	2313	384.84320	286.87540	269.81311	256.90852	250.89866

Compound	CCS (Å^2)	RSD (%)	Family	Formula	LRI_{exp}	F1	F2	F3	F4	F5
etofenprox	189.39	0.15	Pesticides	C25H28O3	2850	183.08031	163.11147	135.08012	107.04888	
fenamiphos	170.80	0.26	Pesticides	C13H22NO3PS	2145	217.00767	260.05020	288.08161	154.04371	234.03425
fenvalerate I	195.56	0.20	Pesticides	C25H22ClNO3	2928	225.07872	167.06223	125.01518		
fenvalerate II	195.42	0.18	Pesticides	C25H22ClNO3	2959	225.07872	167.06223	125.01518		
flucythrinate	200.93	0.10	Pesticides	C26H22NO4	2834	199.09275	157.04589	225.07810	184.03291	107.04973
fludioxonil	147.68	0.03	Pesticides	C12H6F2N2O2	2152	182.04667	127.04094	154.05190	101.03777	
fluquinconazole_F	177.57	0.20	Pesticides	C16H9Cl2FN4O	2671	226.99251	210.00777	306.96011	109.00452	306.96011
heptachlor	155.14	0.27	Pesticides	C10H5Cl7	1888	334.85176	263.90689	229.94573	229.94573	134.97719
heptachlor epoxide a	157.61	0.49	Pesticides	C10H5Cl7O	1993	253.90250	288.87203	182.91690	352.84630	316.86687
heptachlor epoxide b_F	155.39	0.05	Pesticides	C10H4Cl6O	1988	252.89561	216.93737	262.85632	281.89810	299.86389
hexachlorobenzene	134.68	0.22	Pesticides	C6Cl6	1688	246.84414	211.87514	176.90622		
hexachlorobutadiene	131.11	0.12	Pesticides	C4Cl6	1218	189.87158	224.84051	140.90579	154.90283	117.93706
indoxacarb	214.92	0.22	Pesticides	C22H17ClF3N3O7	3030	264.02972	218.04231	203.01879	176.00239	235.04492
isodrin	153.87	0.29	Pesticides	C12H8Cl6	1973	326.90621	290.92993	192.93788		
isopyrazam	181.90	0.16	Pesticides	C20H23F2N3O	2626	159.03643	303.11719	344.15652	262.09729	276.11283
mirex_F	132.83	0.15	Pesticides	C5Cl6	2551	234.84380	164.90553	140.90560	116.90579	
oxyfluorfen	175.37	0.04	Pesticides	C15H11ClF3NO4	2196	300.00266	237.03959	252.03884	315.99803	222.97683
pentachlorobenzene	130.33	0.18	Pesticides	C6HCl5	1508	212.88191	177.91234	176.90495	141.93570	
pentachlorophenol	134.35	0.05	Pesticides	C6HOCl5	1741	164.90602	201.87198	227.86958		
permethrin I	187.55	0.06	Pesticides	C21H20Cl2O3	2687	355.10844	354.10138	319.13202	254.04911	183.08009
permethrin II	187.51	0.36	Pesticides	C21H20Cl2O4	2706	355.10844	354.10138	319.13202	254.04911	183.08009
propethamphos	163.12	0.53	Pesticides	C10H20NO4PS	1779	194.04039	138.01370	238.02977	111.06770	165.98475
propham_F	127.10	0.18	Pesticides	C7H7NO2	1461	120.04478	93.05804	77.03972		
resmethrin	184.54	0.10	Pesticides	C22H26O3	2410	279.17417	171.08027	143.08027	251.14284	323.16385
spiromesifen	196.08	0.38	Pesticides	C23H30O4	2413	272.14071	254.13043	209.13261	231.10198	186.06784
sulprofos	171.60	0.18	Pesticides	C12H19O2PS3	2295	218.96973	156.00603	279.98026	138.99757	96.95136
terbacil_F	125.35	0.18	Pesticides	C5H5N2O2Cl	1804	143.98526	118.00624	87.99616		
tolyfluanid	159.89	0.08	Pesticides	C10H13Cl2FN2O2S2	1996	237.96529	181.07844	137.02828		
trans-chlordane_F	156.60	0.19	Pesticides	C10H5Cl7	2063	265.90322	300.87251	336.84880	229.94470	
α HCH_F	123.82	0.07	Pesticides	C6H3Cl3	1686	145.96846	144.96090	108.98449		

Compound	CCS (Å^2)	RSD (%)	Family	Formula	LRI_{exp}	F1	F2	F3	F4	F5
β HCH_F	123.33	0.11	Pesticides	C6H3Cl3	1735	144.96084	121.06533	108.98458	77.03973	
γ HCH_F	123.49	0.05	Pesticides	C6H3Cl3	1749	144.95973	108.98338	126.99411		
δ HCH_F	123.41	0.08	Pesticides	C6H3Cl3	1807	144.96022	108.98389	74.01545	108.98338	
ehdhp	189.58	0.13	OPFRs	C20H27O4P	2413	251.04711	170.07164	153.06895	233.03620	94.04148

Table S3. Compounds showing CCS deviations between both ionic species $[M+H]^+$ and $M^{+\bullet}$ greater than 2%.

Compound	Formula	CCS in $[M+H]^+$ (Å^2)	CCS in $M^{+\cdot}$ (Å^2)	ΔCCS (%)
tolyfluanid	C8H7NFSCl2	163.11	159.89	2.0
chlorfenson	C12H8Cl2O3S	161.35	158.08	2.1
2-phenylphenol	C12H10O	133.57	130.74	2.2
permethrin II	C21H20O3Cl	191.71	187.51	2.2
atrazine desethyl	C6H10ClN5	138.64	135.59	2.2
tefluthrin	C17H14ClF7O2	176.40	180.48	-2.3
diflufenican	C19H11F5N2O2	188.06	183.86	2.3
propethamphos	C10H20NO4PS	159.32	163.12	-2.3
chlorpyrifos methyl	C7H7Cl3NO3PS	153.47	157.20	-2.4
carfentrazone ethyl	C15H14Cl2F3N3O3	179.06	183.89	-2.6
cypermethrin IV	C22H19Cl2NO3	200.33	195.00	2.7
cyfluthrin IV	C22H18Cl2FNO3	200.44	195.03	2.8
cyfluthrin II	C22H18Cl2FNO3	200.53	195.07	2.8
triflumizole	C15H15ClF3N3O	174.71	169.73	2.9
chlorfenvinfos	C12H14Cl3O4P	167.00	172.40	-3.1
metconazole	C17H22ClN3O	168.67	174.41	-3.3
cypermethrin III	C22H19Cl2NO3	199.97	193.55	3.3
cypermethrin II	C22H19Cl2NO3	199.97	193.55	3.3
cyfluthrin III	C22H18Cl2FNO3	200.53	193.45	3.7
vinclozolin	C12H9Cl2NO3	162.10	156.37	3.7
BTBPE	C14H8Br6O2	189.07	182.34	3.7
fenoxycarb	C17H19NO4	179.77	173.21	3.8
aldrin	C12H8Cl6	155.10	161.65	-4.1
chinomethionate	C10H6N2OS2	143.66	137.84	4.2
endosulfan ether	C9H6Cl6O	152.53	146.19	4.3
quintocene	C6Cl6NO2	146.24	139.56	4.8
indoxacarb	C22H17ClF3N3O7	203.97	214.92	-5.1
endrin	C12H8Cl6O	163.84	155.20	5.6
cyanophos	C9H10NO3PS	154.25	146.07	5.6
chlorothalonil	C8Cl4N2	150.03	140.00	7.2
endosulfan sulfate	C9H6Cl6O4S	170.87	156.41	9.2

Table S4. Screening of multiclass pesticides in river water samples, fruits and vegetable.

Sample	Compound	Mass error (ppm)							ΔCCS (%)
		$M^{+\cdot}$	$[M+H]^+$	F1	F2	F3	F4	F5	
Apple	fludioxonil	3.4		-5.3*	-4.3	-3.2			-0.49
Apple	thiabendazole		3.3	-9.1*					1.31
Apple	mephosfolan		3.6	-8.4*					1.02
Apple	bromopropylate_F	4.6		-3.0					-0.37
Apple	fenhexamid		2.0	0.0					0.27
Apple	atrazinedesisopropyl		4.2						-0.77
Apple	azoxystrobin		2.8						0.17
Apple	bromuconazole I		1.5						1.29
Apple	bromuconazole II		1.5						0.74
Apple	fenaminphos	2.8							0.11
Apple	fenoxycarb		2.4						0.57
Apple	fenoxycarb		2.4						0.57
Apple	isopyrazam	1.6							0.21
Carrot	DDE	4.0							0.26
Carrot	fludioxonil	2.9							0.13
Cauliflower	fludioxonil	2.6		-4.8	-4.4	-7.5*			-0.34
Cauliflower	difeconanzole_F	4.6		-3.6					-0.12
Cauliflower	propiconazole		2.0	-7.7*					1.03
Cauliflower	cyproconazole		3.7						0.83
Cauliflower	cyprodinil		-1.0						0.44
Cauliflower	fenarimol		5.9*						0.00
Cauliflower	fenhexamid		3.1						0.08
Cauliflower	thiabendazole		7.6*						0.16
Cucumber	cyprodinil		0.3	-4.3	-3.2	-9.1*		6.1*	0.40
Cucumber	fludioxonil	3.1		-6.0*	-6.1*	-3.6			-0.04
Cucumber	difeconanzole_F	4.0		1.2					0.05
Cucumber	azoxystrobin		1.9						0.30
Cucumber	propiconazole		3.2						0.68
Green pepper	azoxystrobin		1.4						0.27
Green pepper	cyprodinil		0.6						0.13
Green pepper	difeconanzole_F	3.1							-0.75
Green pepper	fludioxonil	2.9							-0.02
Orange	fludioxonil	3.3		-8.2*	-6.0*	-7.7*			0.11
Red pepper	cyprodinil		0.5						0.72
Red pepper	fludioxonil	2.5							0.02
Tomato	fludioxonil	2.5		-6.2*	-6.0*	-4.1			0.10
Tomato	difeconazole_F	3.0		1.2	1.1				-0.12
Tomato	propiconazole		2.2	2.0	1.9				0.57
Tomato	azaconazole		2.8	3.8					1.00
Tomato	bromopropylate_F	4.6		-7.7*					-0.27
Tomato	cyanazine		0.7	0.3					0.28
Tomato	difluencian	4.1		-4.4					-0.39

Sample	Compound	Mass error (ppm)							ΔCCS (%)
		$M^{+\cdot}$	$[M+H]^+$	F1	F2	F3	F4	F5	
Tomato	metalaxyl		1.5		-0.6				0.92
Tomato	44dichlorobenzophenone		-3.9						0.53
Tomato	azoxystrobin		3.0						0.01
Tomato	cyproconazole		1.3						1.01
Tomato	cyprodinil		2.0						0.40
Tomato	DDE	3.3							0.09
Tomato	difenoconazole		2.8						1.40
Tomato	EPN		3.8						0.38
Tomato	famphur		3.5						0.50
Tomato	fenarimol		3.0						0.70
Tomato	fenhexamid		2.0						0.29
Tomato	fenpropimorph		-0.2						0.66
Tomato	isopyrazam	2.9							0.16
Tomato	metconazole		2.7						1.12
Tomato	tebuconazole		2.4						0.81
Tomato	terbutryn		1.8						0.89
Tomato	thiabendazole		4.3						0.81
Tomato	triadimefon		1.6						0.78
RW17	fludioxonil	-0.6		-0.8	0.9	1.8			-0.18
RW17	propiconazole		0.5	2.0	1.9				-1.04
RW17	thiabendazole		-3.4	2.6	-0.2				0.03
RW17	terbutryn		-1.6	-1.5					0.30
RW17	diazinon		1.8						0.70
RW18	fludioxonil	0.2		-4.3	-0.2	-2.0			0.03
RW18	terbutryn		-0.9	-1.5					0.13
RW18	thiabendazole		-2.8		-0.1				0.18
RW18	diazinon		-1.4						0.26
RW18	propiconazole		2.2						-0.13
RW19	terbumeton		-0.4	-3.1					-0.17
RW19	terbutryn		-3.9	-1.5					0.39
RW19	propiconazole		-0.5						0.20
RW19	terbuthylazine		-5.0						0.87

*mass errors above 5 ppm.

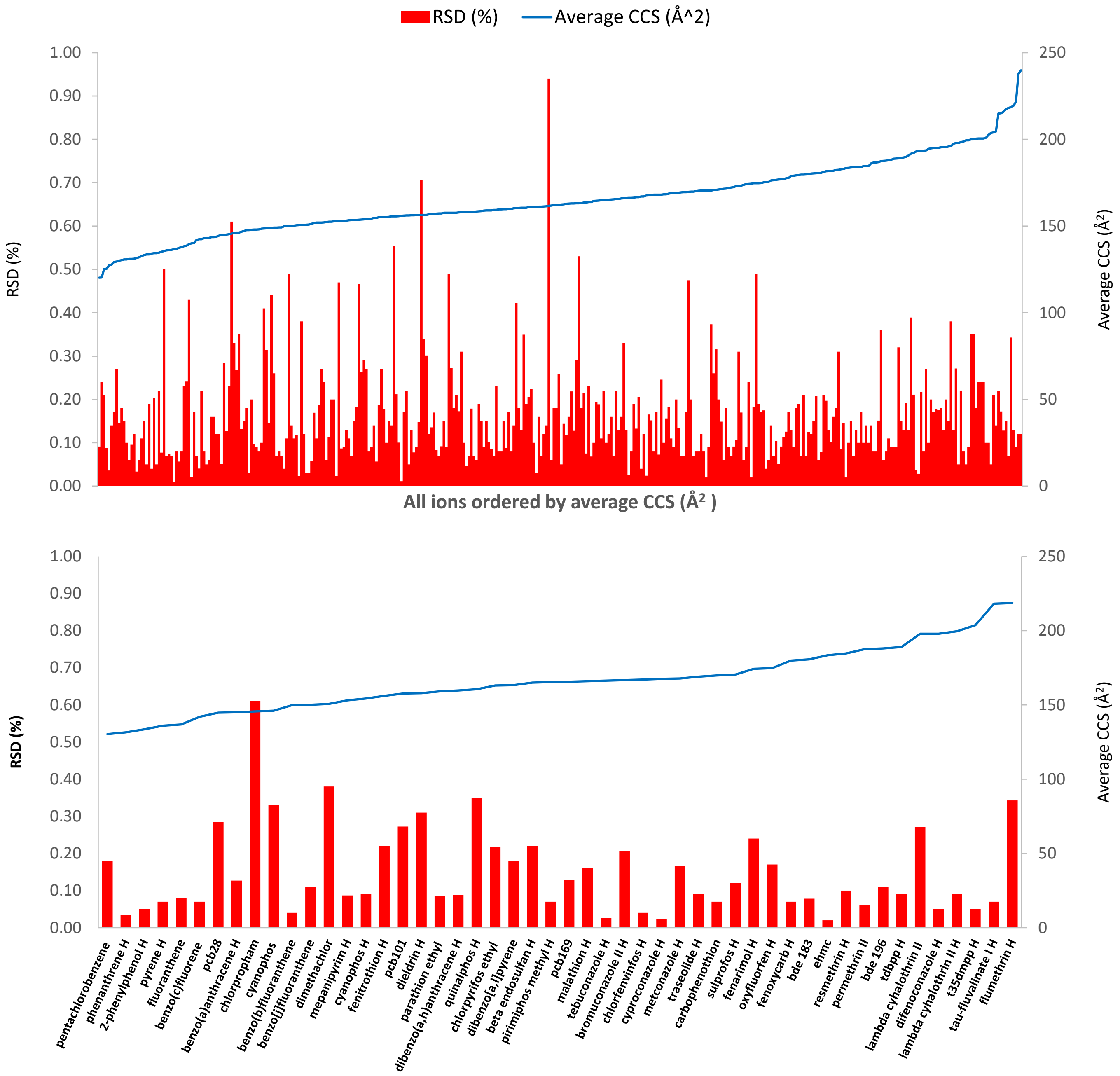

Figure S1. Average CCS value (n=6) and Relative Standard Deviation (RSD) for all ionic species (above) and for 50 randomly selected ion species, the "H" refers to $[M+H]^+$ whereas the absence of it refers to $M^{+\bullet}$ (bellow). In both graphs, ion species are ordered by means of average CCS values on the x-axis.

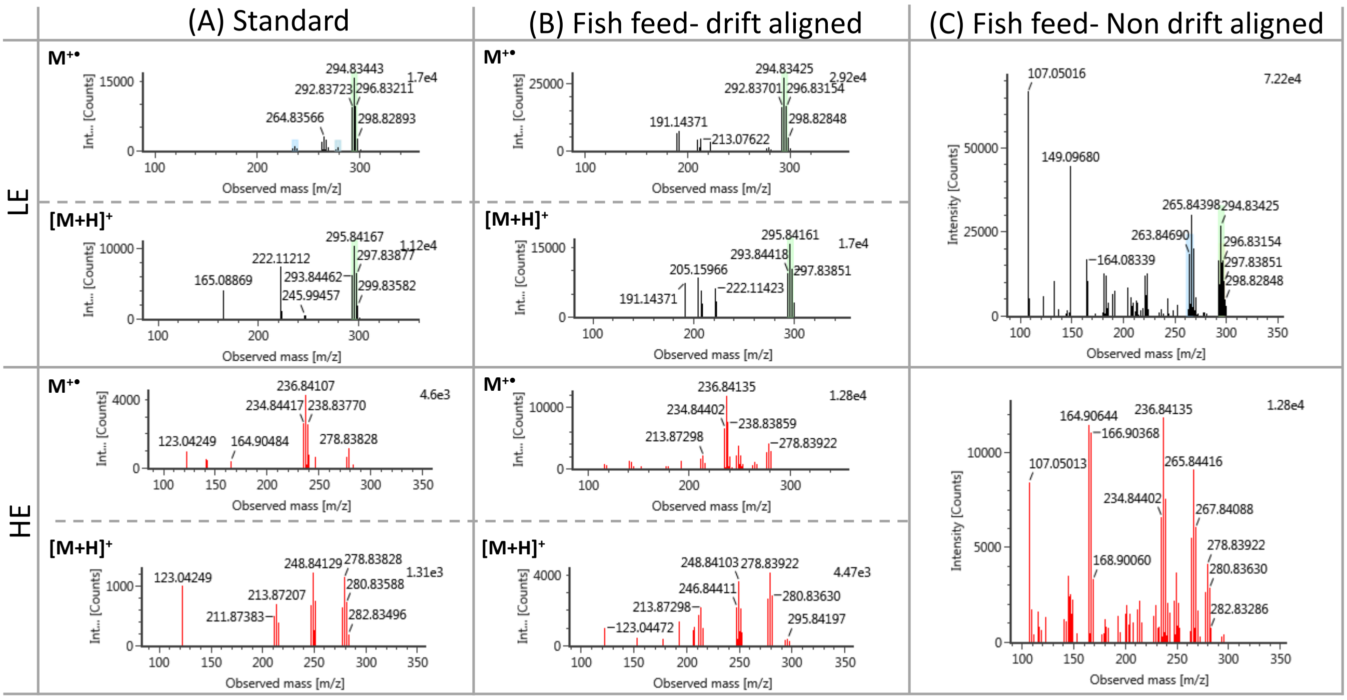

Figure S2. Comparison of HRMS spectra for quintozene in analytical reference standard solution at 10 µg L^{-1} (A), DT aligned data in fish feed sample spiked at 10 µg L^{-1} (B) and non-DT aligned data of the same finding in the same spiked fish feed sample (C). Low energy (LE) and high energy (HE) spectra are shown for both species [M+H]$^{+}$ and M$^{+\bullet}$

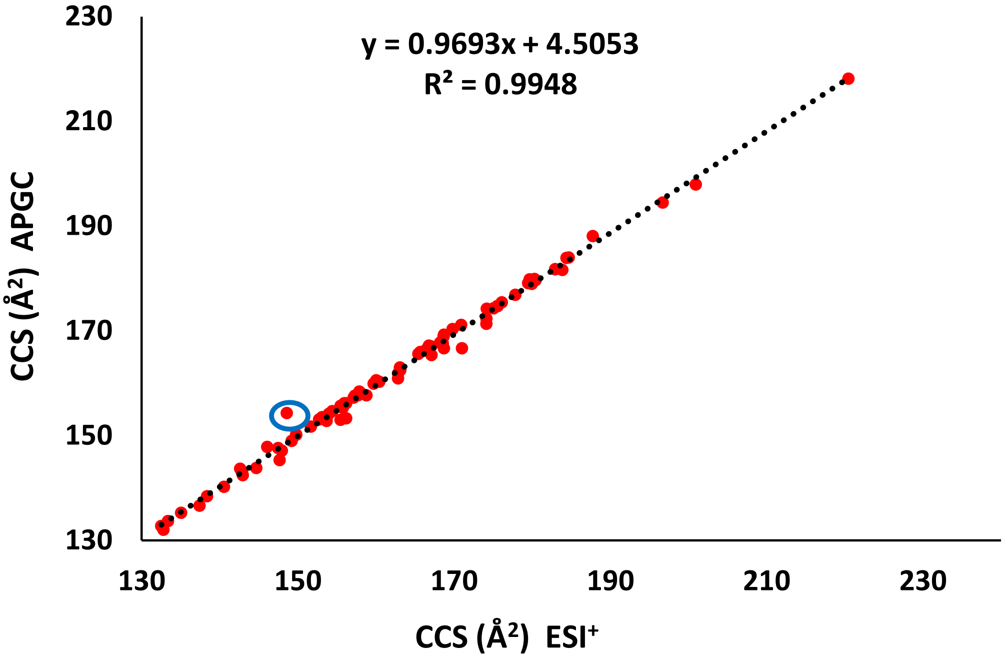

Figure S3. Regression plot of the CCS values for protonated molecules from GC-APCI and LC-ESI. The blue circle surrounds cyanophos.

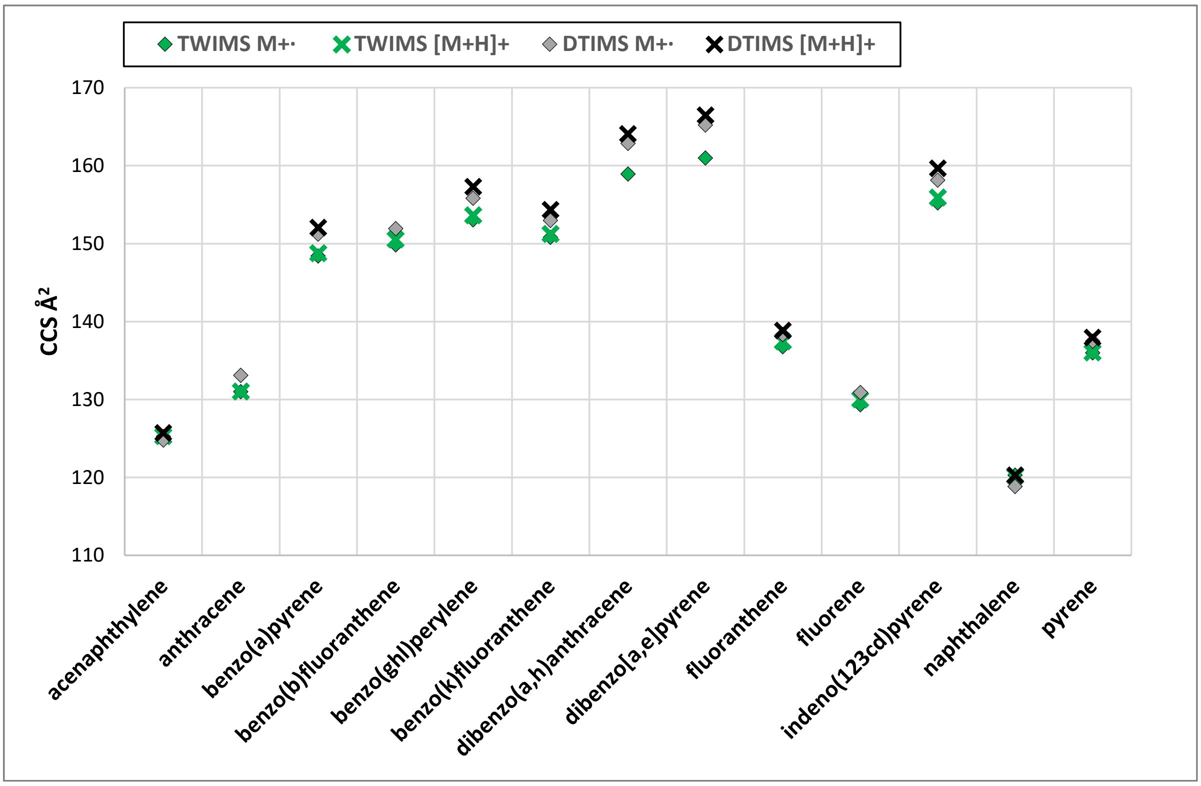

Figure S4. Comparison of CCS values (Å²) obtained by different IMS technologies for PAHs. CCS^{DTIMS} were taken for the work of Zheng et al.[5] and CCS^{TWIMS} from the library of the current work.

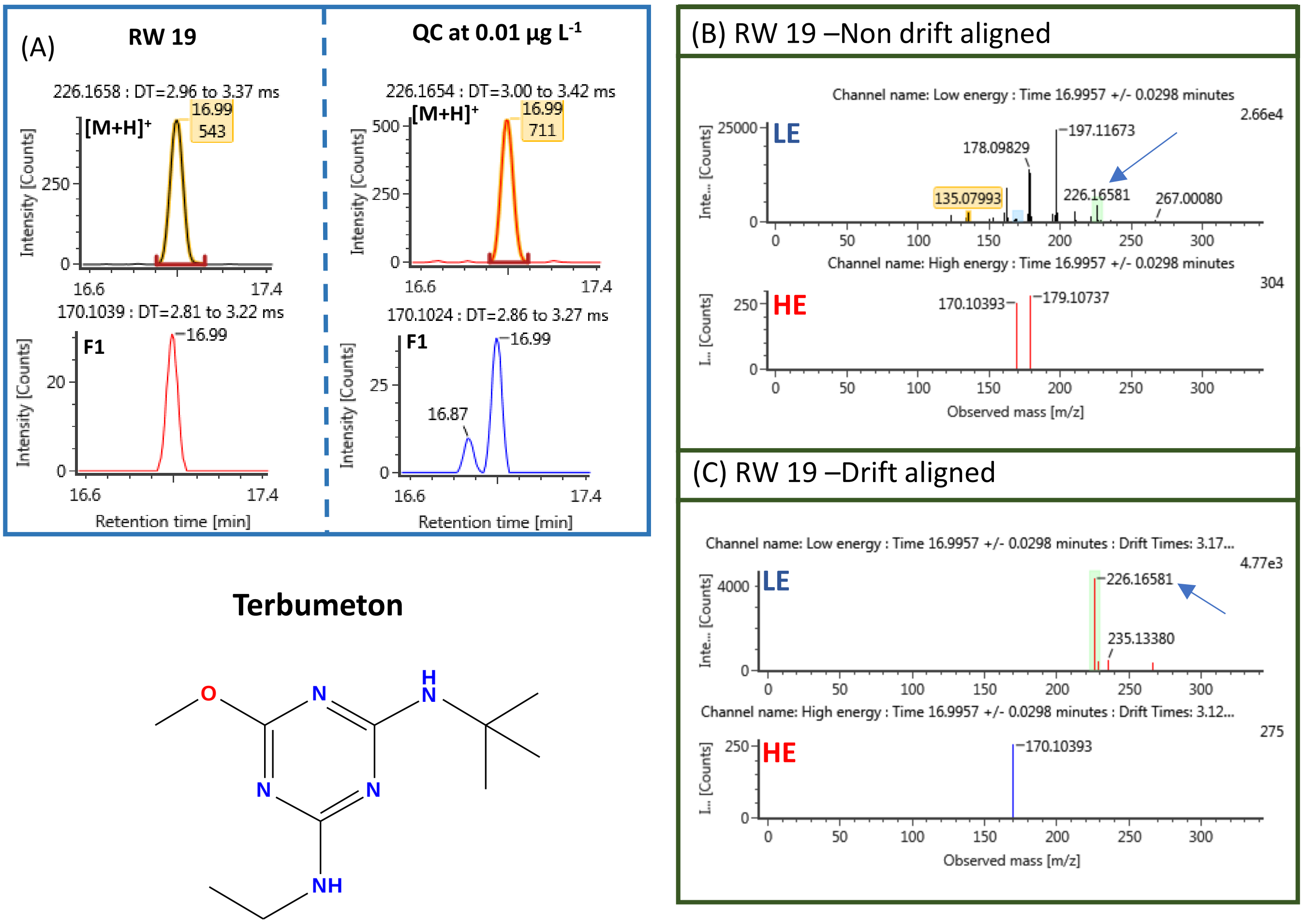

Figure S5. Identification of terbumeton in surface water. (A) Extracted-ion chromatogram (XIC) (mass window ± 0.01Da) showing a positive finding in surface water. (B) Conventional LE (top) and HE (bottom) mass spectra without IMS drift time alignment. (C) LE (top) and HE (bottom) mass spectra with IMS drift time alignment.

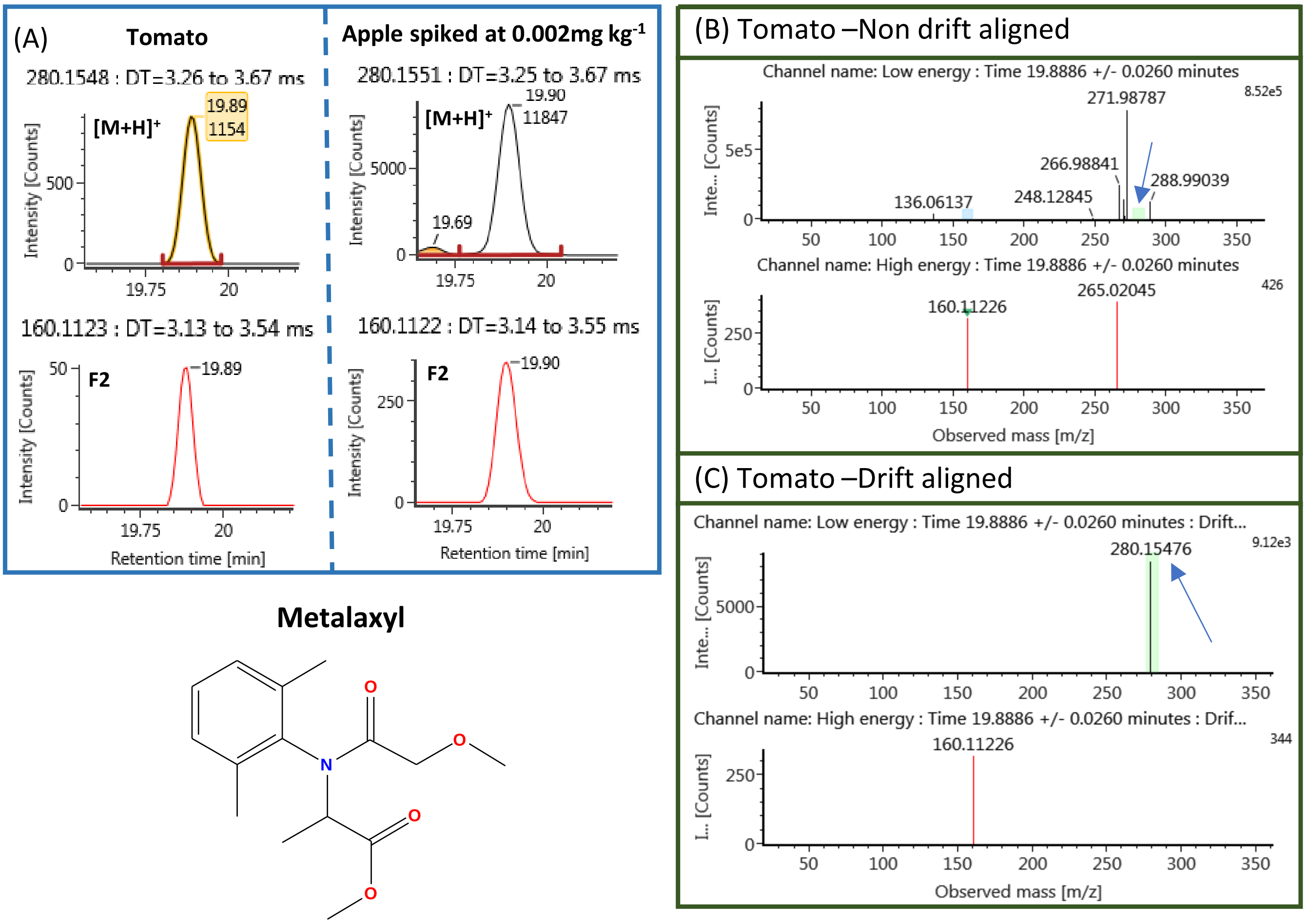

Figure S6. Identification of metalaxyl in tomato. (A) XIC (mass window ± 0.01Da) showing a positive finding in tomato. (B) Conventional LE (top) and HE (bottom) mass spectra without IMS drift time alignment. (C) LE (top) and HE (bottom) mass spectra with IMS drift time alignment.

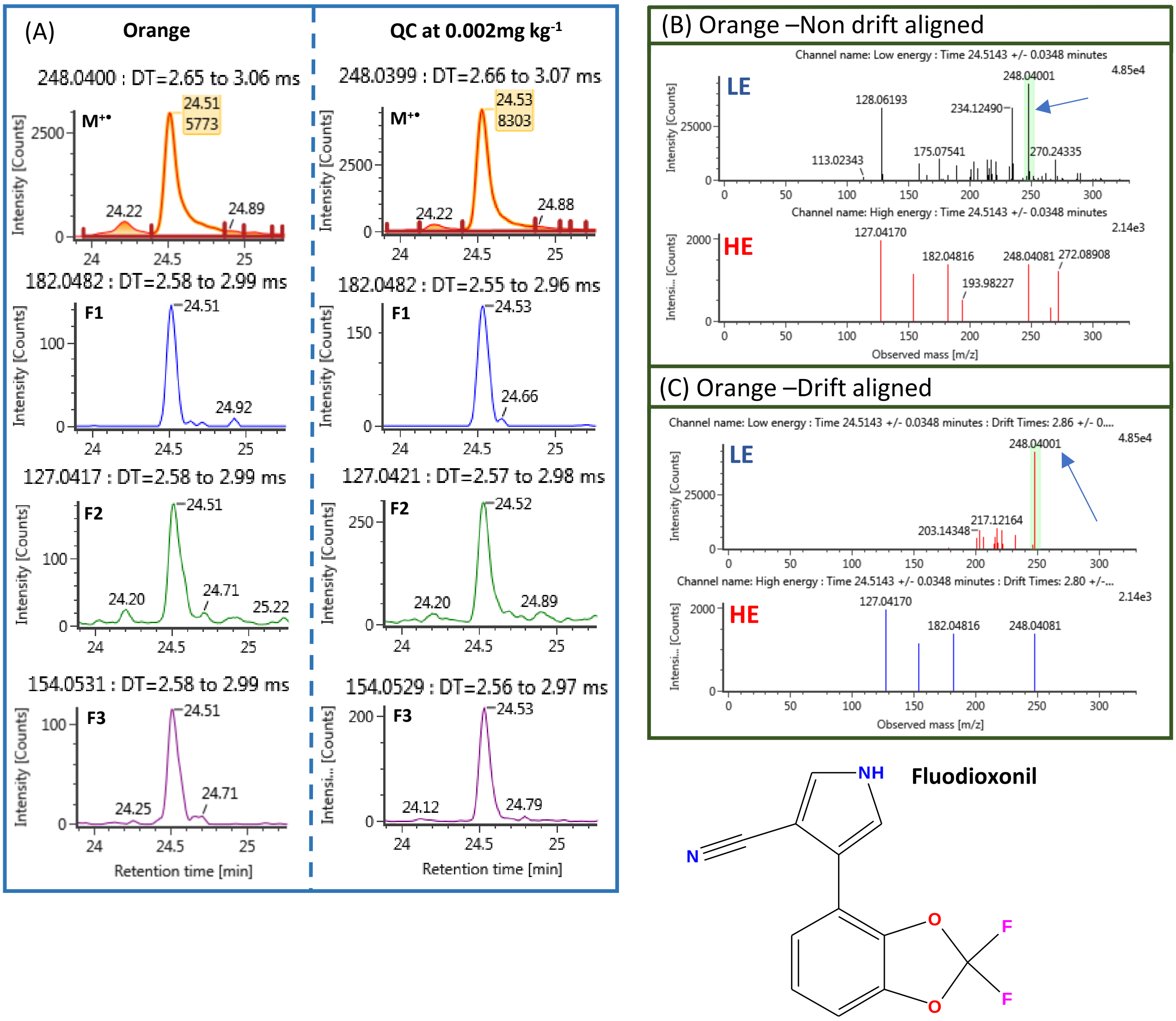

Figure S7. Identification of fludioxonil in orange. (A) XIC (mass window ± 0.01Da) showing a positive finding in orange. (B) Conventional LE (top) and HE (bottom) mass spectra without IMS drift time alignment. (C) LE (top) and HE (bottom) mass spectra with IMS drift time alignment.

REFERENCES

(1) Bijlsma, L.; Pitarch, E.; Hernández, F.; Fonseca, E.; Marín, J. M.; Ibáñez, M.; Portolés, T.; Rico, A. Ecological Risk Assessment of Pesticides in the Mijares River (Eastern Spain) Impacted by Citrus Production Using Wide-Scope Screening and Target Quantitative Analysis. *J. Hazard. Mater.* **2021**, *412, 125277*. https://doi.org/10.1016/j.jhazmat.2021.125277.

(2) Portolés, T.; Garlito, B.; Nácher-Mestre, J.; Berntssen, M. H. G.; Pérez-Sánchez, J. Multi-Class Determination of Undesirables in Aquaculture Samples by Gas Chromatography/Tandem Mass Spectrometry with Atmospheric Pressure Chemical Ionization: A Novel Approach for Polycyclic Aromatic Hydrocarbons. *Talanta* **2017**, *172*, 109–119. https://doi.org/10.1016/j.talanta.2017.05.025.

(3) Portolés, T.; Mol, J. G. J.; Sancho, J. V.; López, F. J.; Hernández, F. Validation of a Qualitative Screening Method for Pesticides in Fruits and Vegetables by Gas Chromatography Quadrupole-Time of Flight Mass Spectrometry with Atmospheric Pressure Chemical Ionization. *Anal. Chim. Acta* **2014**, *838*, 76–85. https://doi.org/10.1016/j.aca.2014.06.006.

(4) European Commission. Guidance Document on the Analytical Quality Control and Method Validation Procedures for Pesticide Residues in Food and Feed. No SANTE/12682/2019. *Eur. Comm. Heal. Consum. Prot. Dir.* **2019**, *12682*, 3–49.

(5) Zheng, X.; Dupuis, K. T.; Aly, N. A.; Zhou, Y.; Smith, F. B.; Tang, K.; Smith, R. D.; Baker, E. S. Utilizing Ion Mobility Spectrometry and Mass Spectrometry for the Analysis of Polycyclic Aromatic Hydrocarbons, Polychlorinated Biphenyls, Polybrominated Diphenyl Ethers and Their Metabolites. *Anal. Chim. Acta* **2018**, *1037*, 265–273. https://doi.org/10.1016/j.aca.2018.02.054.
